# Colour by design: tuning the solid-state emission of coronene bisimide by tailored matrices

**DOI:** 10.1039/d6sc02467j

**Published:** 2026-05-28

**Authors:** Simon Soldner, Ömer E. Öçal, Kazutaka Shoyama, Dominik Horneber, Johannes Düreth, Sven Höfling, Sebastian Klembt, Matthias Stolte, Frank Würthner

**Affiliations:** a Center for Nanosystems Chemistry (CNC), Universität Würzburg Theodor-Boveri-Weg Würzburg 97074 Germany frank.wuerthner@uni-wuerzburg.de; b Institut für Organische Chemie, Universität Würzburg Am Hubland Würzburg 97074 Germany; c Julius-Maximilians-Universität Würzburg, Physikalisches Institut, and Würzburg-Dresden Cluster of Excellence ctd.qmat, Lehrstuhl für Technische Physik Am Hubland Würzburg 97074 Germany; d Julius-Maximilians-Universität Würzburg, Physikalisches Institut, and Würzburg-Dresden Cluster of Excellence ctd.qmat, Lehrstuhl für Experimentelle Physik 1 Am Hubland Würzburg 97074 Germany

## Abstract

A photofunctional coronene bisimide (CBI) has been equipped with two bulky terphenyl imide substituents that control self-assembly, restricting it to the formation of well-defined dimers with a remarkably high association constant of *K* = 3.0 × 10^5^ M^−1^ in methylcyclohexane/1,1,2,2-tetrachloroethane (TCE). Further, the electron-poor CBI acts as a versatile supramolecular host capable of forming charge-transfer (CT) complexes with various electron-rich guest molecules, including perylene, triphenylene, and 3,6-diiodocarbazole with binding affinities up to *K* = 10^3^ M^−1^ in TCE. As shown in this article, the variety of supramolecular assemblies formed by CBI enables a unified strategy for emission colour tuning over a wide spectral range. In the crystalline solid-state, the photoluminescence can be tuned from green up to the near-infrared region through either monomer-like or dimer-like polymorphs, or guest binding. Further, the external heavy-atom effect of 3,6-diiodocarbazole allows for dual emission of thermally activated delayed fluorescence and phosphorescence, making the cocrystal with diiodocarbazole the first example of a CBI cocrystal with delayed emission. This study presents a general design strategy for emission tuning of CBIs, which is expected to be extendable to a variety of luminescent host systems.

## Abstract 2

The photoluminescence of a shielded coronene bisimide can be tuned in the solid state *via* monomer- or dimer-like crystallization or by guest encapsulation, spanning visible fluorescence to NIR phosphorescence with quantum yields up to 19%.

## Graphical abstract

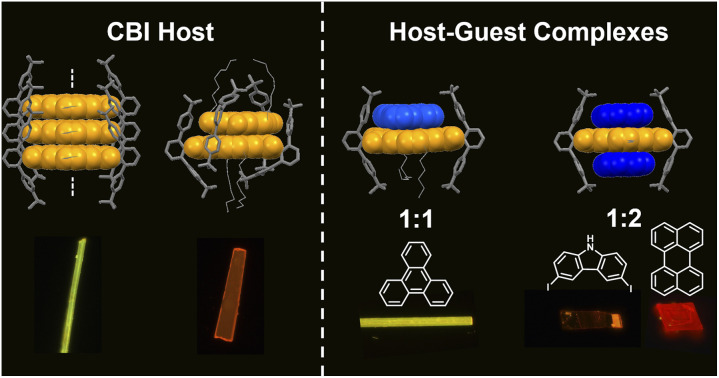

## Introduction

Materials with tunable emission properties are in demand for various applications including organic light-emitting diodes (OLEDs),^1^ organic lasers,^2,3^ and bioimaging.^4^ The respective materials are often based on transition or rare earth metal complexes which are disadvantageous from the perspective of abundancy and sustainability.^5,6^ In this regard it is promising that recent research on purely organic systems demonstrated emission colour tuning through polymorphism,^7,8^ guest encapsulation^9^ or for carbon dots.^10,11^ Further, as a particularly promising approach, we and others identified tailored supramolecular host–guest systems making use of donor–acceptor interactions for tuning emission properties.^12–14^ Different from conventional donor–acceptor π-stacks whose emission originates purely from excited singlet states (exciplexes), this research established the benefits originating from the involvement of triplet states for the expansion of the wavelength range and the increase of photoluminescence quantum yield by additional photon fluxes originating from delayed fluorescence or phosphorescence. A particularly compelling example has been introduced by George and co-workers for solutions and cocrystals of a simple pyromellitic bisimide.^15^ By simple dissolution of pyromellitic bisimide in various electron-donating solvents the emission colour of the singlet charge-transfer (^1^CT) state could be tuned from 420 nm (tetrahydrofuran) to 500 nm (tetramethylbenzene). Furthermore, by cocrystallization with diiodobenzenes the wavelength range could be expanded up to 560 nm due to the activation of phosphorescence from the triplet CT (^3^CT) state.^15^

What remains as a limitation, however, is the fact that the weak non-covalent interaction strength in donor–acceptor complexes of small aromatic compounds^16^ can only be used either with a large excess of one component, *i.e.* applied as a solvent, or in cocrystals, the latter requiring proper size match between the two components.^17^ A solution to this problem can be realized by larger aromatic surfaces which provide sufficient binding strength to form tightly bound donor–acceptor complexes in solution as well as in amorphous matrices as applied in OLED research.^14,18–20^ With few exceptions, however, this research has not yet demonstrated broad wavelength tunability but was primarily focused on the exploration of the various photophysical processes upon photoexcitation of pre-assembled donor–acceptor pairs, *i.e.* emission from exciplex states, thermally activated delayed fluorescence (TADF), and phosphorescence.^21^

To demonstrate the concept of emission colour tuning over a wider spectral range by means of triplet state activation in donor–acceptor complexes, coronene appeared as an interesting candidate due to its triplet accessibility, which affords phosphorescence and TADF emission.^22–26^ Polymorphism has also been described for coronene,^27^ and its large π-surface makes it a promising host candidate for guest complexation. Compared to coronene, electron-poor coronene bis(dicarboximides) (CBIs) exhibit a larger π-scaffold which together with the lower electron density allows for more effective complexation of various aromatic guest molecules. Furthermore, the thoughtful choice of bulky imide substituents might be used to design desirable host–guest complexes. However, unlike coronene, there are only a few examples of CBIs exhibiting such emission features originating *via* the involvement of the triplet states.^19,28–31^ In 2014, Fukuzumi, Hasobe and co-workers reported a study on coronenes being equipped with one up to four imide groups. For several derivatives high quantum yields were reported for intersystem crossing (ISC), however, only little information on the phosphorescence observed in nitrogen-purged frozen 2-methyl tetrahydrofuran at 77 K was provided.^28^ Three years later, the same group focused on bisimides but only reported lifetimes of the locally excited triplet state (^3^LE).^29^ At the same time, Hariharan and co-workers demonstrated that core-twisting of annulated CBI derivatives enhances the triplet formation yield. However, phosphorescence emission was only observed at 77 K.^30^ Then in 2024, Zhao *et al.* reported the rapid ISC and triplet lifetimes for a CBI with nanohoops attached to the chromophore, but likewise did not report phosphorescence spectra.^31^ In the same year, our group reported a CBI cyclophane with high binding affinities to various polycyclic aromatic hydrocarbon (PAH) guest molecules in solution. For this system, the guest molecules could activate different emission pathways *via* the newly formed ^1^CT states. Binding guest molecules containing heavy atoms such as platinum acetylacetonate or 1,8-dibromonaphthalene even enabled dual emission involving TADF and room-temperature phosphorescence (RTP).^19^

Following this precedent work, herein we report our results for a new CBI derivative bearing sterically demanding imide substituents for which we could accomplish emission colour tuning from the green up to the near-infrared (NIR) region by judicious choice of the surrounding matrix. This desirable feature could be achieved by the strong and well-defined supramolecular binding of the enlarged π-scaffold of CBI, compared to conventional smaller donor–acceptor complexes, thereby enabling self-aggregation and complexation with different guest molecules. A maximum photoluminescence (PL) quantum yield (*Φ*_PL_) of 19% was thereby reached for the dimer containing crystal of CBI 1. Additionally, TADF and RTP emission were activated by the external heavy-atom effect from cocrystal components. Importantly, because these systems form structurally defined, non-dynamic supramolecular assemblies, they also exhibit polarization-dependent photoluminescence properties. Overall, this work demonstrates that rational molecular design enables broad-range emission tuning in donor–acceptor systems through controlled supramolecular assembly.

## Results and discussion

### Synthesis, characterization, and crystallization

**Fig. 1:**
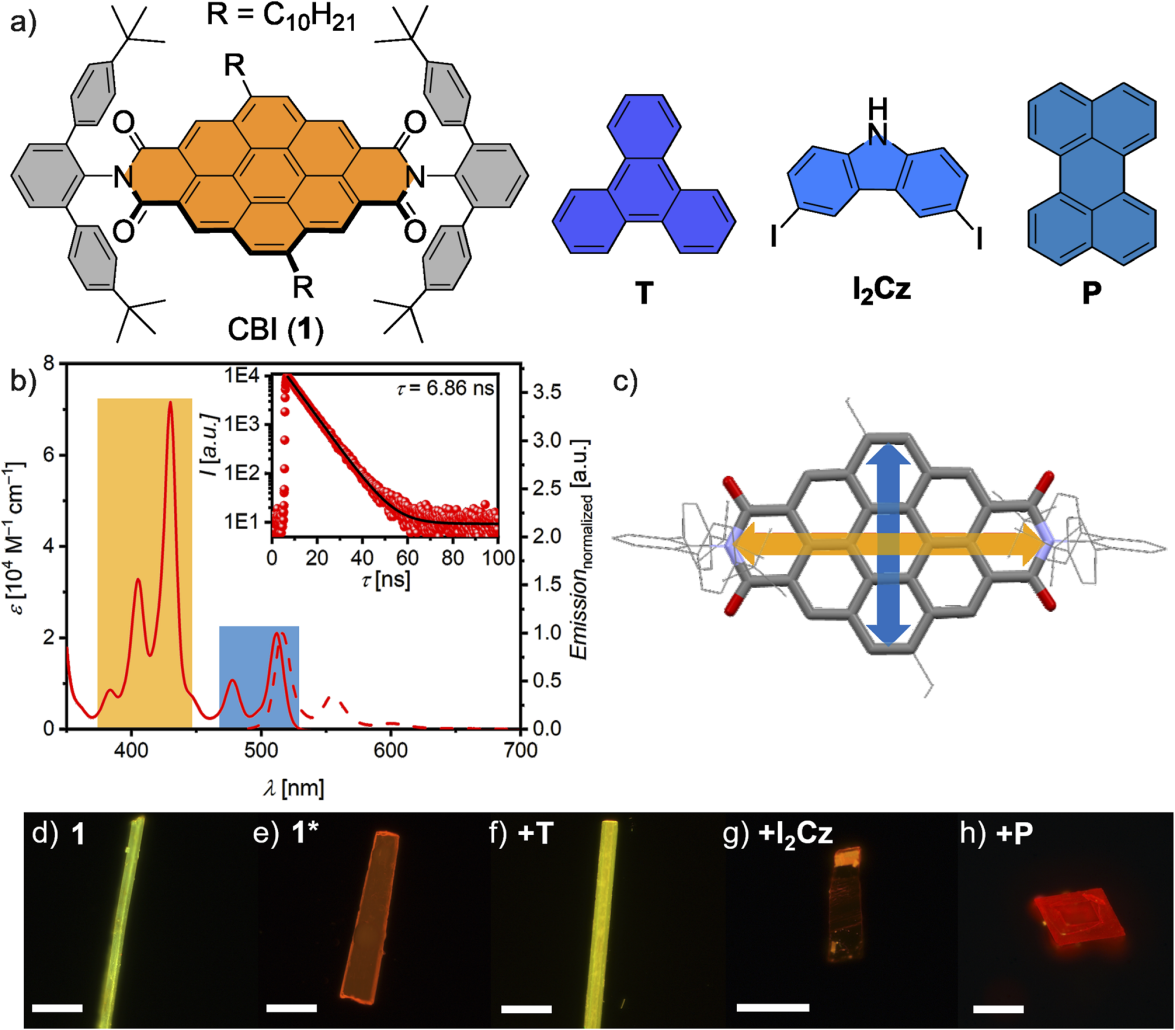
(a) Chemical structures of the investigated shielded host molecule CBI 1 and the guest molecules T, I_2_Cz and P. (b) UV/vis absorption (solid) and scaled emission (dashed) spectra of CBI 1 in CHCl_3_ solution (*c*_0_ = 1.2 × 10^−5^ M and 2.7 × 10^−7^ M) at room temperature. The emission was detected after excitation at 476 nm. Inset depicts lifetime measurement (red circles) after excitation at 479.7 nm while detecting the respective maximum. The mono-exponential fit is also shown (black line) as well as the lifetime is given. (c) Illustration of the S_0_–S_1_ (blue arrow) and S_0_–S_2_ (gold arrow) transition dipole moments of CBI. Fluorescence microcopy images of microcrystals of two single crystals of 1 (d and e) and cocrystals with T (f), I_2_Cz (g) and P (h) on Si/SiO_2_ substrates at 298 K upon UV irradiation. The scale bars equal 100 µm.

CBI 1 has been designed as a supramolecular host bearing sterically demanding *meta*-terphenyl imide substituents and two alkyl chains attached to the coronene core unit. The latter induce higher solubility of the large and planar CBI π-scaffold (Fig. 1a) while the *meta*-terphenyl groups bearing *tert*-butyl substituents in the *para*-position suppress molecular aggregation and promote the uptake of planar (hetero-)aromatic guest molecules.^32–34^ As guest molecules we chose triphenylene (T), perylene (P), and 3,6-diiodocarbazole (I_2_Cz) (Fig. 1a). CBI 1 was synthesized in 31% yield *via* an optimized imidization procedure,^32,35^ using precursor molecules synthesized according to the literature (Schemes S1 and S2, SI).^36–38^ CBI 1 was characterized using high-resolution mass spectrometry (HRMS), as well as ^1^H-NMR, UV/vis, and PL spectroscopy and X-ray analysis (see SI for details).

The UV/vis absorption spectrum of 1 in chloroform (CHCl_3_) solution is typical of CBI, with well-resolved vibronic S_0_–S_1_ absorption at around 510 nm (*λ*_max_) and an S_0_–S_2_ transition at around 430 nm with extinction coefficients (*ε*_max_) of around 20 000 and 70 000 M^−1^ cm^−1^, respectively (Fig. 1b and Table S1, SI).^19^ With regard to the later discussed excitonic coupling among CBIs in crystalline samples it is important to note the orthogonal arrangement of the transition dipole moments (*µ*_eg_) for these two electronic transitions (Fig. 1c) and the fact that the lowest energy transition (S_1_) is polarized orthogonal to the *N*,*N*′-axis,^39^ a feature that distinguishes CBIs from the related perylene bis(dicarboximides) (PBIs). The emission spectrum of CBI 1 shows a mirror-image relationship to the absorption bands with a fluorescence quantum yield of 31%, a small Stokes shift (Δ*ṽ*_Stokes_ = 151 cm^−1^) and a decay time of 6.9 ns (Fig. 1b).

Single crystals of pristine CBI 1 and cocrystals with P, T, and I_2_Cz suitable for X-ray analysis were grown by slow diffusion of an anti-solvent into the solution of CBI 1. Two different single-crystals of pristine CBI 1 could be grown from CHCl_3_ (*c*_0_ = 10^−3^ M) and iodobenzene solutions (IBz; *c*_0_ = 10^−3^ M) with diffusion of methanol as anti-solvent. For the cocrystals, either toluene (P and T) or CHCl_3_ (I_2_Cz) was used, with methanol (P and T) or *n*-hexane (I_2_Cz) slowly diffusing into the stock solution of CBI 1 (*c*_0_ = 10^−3^ M). The ratio to 1 and guest in the respective solutions varied between 1 : 2 (P and T) and 1 : 4 (I_2_Cz). Suitable crystals of the five (co)crystals of CBI 1 were handpicked, deposited on an Si/SiO_2_ quartz substrate and investigated with a (PL) polarizing optical microscope (POM) under white light (halogen lamp) and UV light irradiation (Fig. 1d–h and S1, SI). The crystals, as well as their emission colour, range from green to deep red. Unexpectedly, two different crystals were obtained from pristine CBI from CHCl_3_ or IBz, respectively, emitting either green, similar to the monomer in solution, or orange light.

**Fig. 2:**
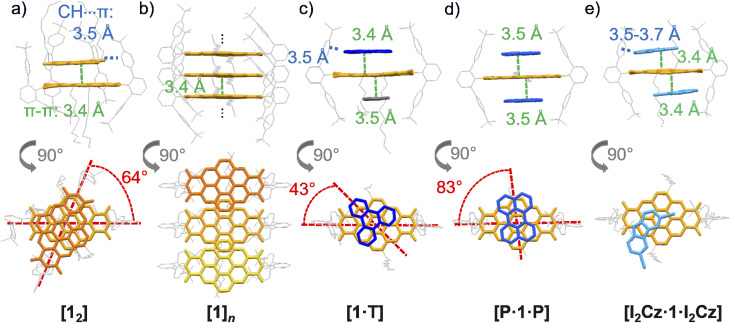
Single-crystal (a and b) and cocrystal structures of the sterically shielded CBI 1 (yellow-orange) with guest molecules (blue) (c) T, (d) P and (e) I_2_Cz shown in side-view (top) and top-view (bottom). The CBI chromophore and guest molecules are displayed in capped sticks, whereas the imide substituents and the substituents at the CBI core are displayed in wireframe (grey). The single-crystals either form separated dimers (a) [1_2_] or (b) a lateral-displaced slip-stacked alignment [1]*_n_*. The cocrystals form (c) either 1 : 1 complex, [1·T] including a toluene molecule (grey), or 1 : 2 complexes, (d) [P·1·P] and (e) [I_2_Cz·1·I_2_Cz]. The distances between the respective *π*-surfaces are shown in green, the C–H⋯π and N–H⋯π distances in blue (side-view), as well as the angle between the molecules in red (top-view). Molecular disorder (b–e) and solvent molecules (b, c and e) are omitted for clarity.

The molecular arrangement of host 1 with and without complexation of guest molecules within the five (co)crystals is illustrated in Fig. 2 (Table S2–S6, SI). The orange emissive single crystal is built up from tightly packed (π–π-distances of 3.4 Å) units of isolated dimers 1_2_ (Fig. 2a and S2, SI) which are rotationally displaced by 64°. Similar supramolecular interactions as for the previously mentioned PBI, like C–H⋯π interactions at 3.5 Å between chromophore and *meta*-terphenyl imide substituent, can be observed.^32^ The crystal obtained from CHCl_3_ also provides proof to the dimerization observed in solution (*vide infra*). In contrast, in the yellow-green-emissive crystal obtained from IBz, the shielded CBI 1 adopts an extended laterally slipped-stack arrangement of parallelly oriented CBI molecules at a distance of 3.4 Å. The centre-to-centre distance is accordingly enlarged 6.8 Å with a lateral displacement of 30° (Fig. 2b and S3, SI).

For all cocrystals, the CBI receptor unit is embedded between two aromatic guest molecules. For the cocrystal with T, CBI is stacked at a distance of 3.4 Å and at a rotation angle of 43° to T whilst its second π-surface is in contact to a π-stacked toluene molecule at a distance of 3.5 Å (Fig. 2c and S4, SI). Thus, despite a 1 : 2 ratio for CBI 1 : T in the crystallization solution, only one T binds in the crystalline solid state. A possible explanation for this observation could be an allosteric effect.^40^ The two solubilizing decyl alkyl chains at the CBI core point both to the other side of the bound T, effectively blocking the π-surface of the CBI and narrowing the cavity. Thus, binding of a second T might be suppressed, and the cavity is instead filled with the smaller toluene molecule. For the other two cocrystals – in accordance with complexation study in solution, *vide infra* – isolated 1 : 2 complexes are obtained for the cocrystal with P (1 : 2). Here a singular molecule 1 is surrounded by two P molecules on both sides of the chromophore's surface. Thus, the two components stack at a distance of 3.5 Å with the long axis of P rotated 83° relative to the long axis of the CBI (Fig. 2d and S5, SI). In the cocrystal with the smallest guest I_2_Cz, one I_2_Cz molecule binds on each side of the CBI π-surface at a distance of 3.4 Å. Additionally, the CBI chromophore is surrounded by two additional I_2_Cz molecules forming hydrogen bonds between the I_2_Cz's N–H and the CBI's C=O moieties with a distance of 2.0 Å (Fig. 2e and S6, SI). Therefore, five different crystals of sterically shielded 1 with and without molecules of different donor abilities could be grown: two single crystals of pristine CBI 1 and three cocrystals containing different guest molecules.

### Aggregation and complexation studies

**Fig. 3:**
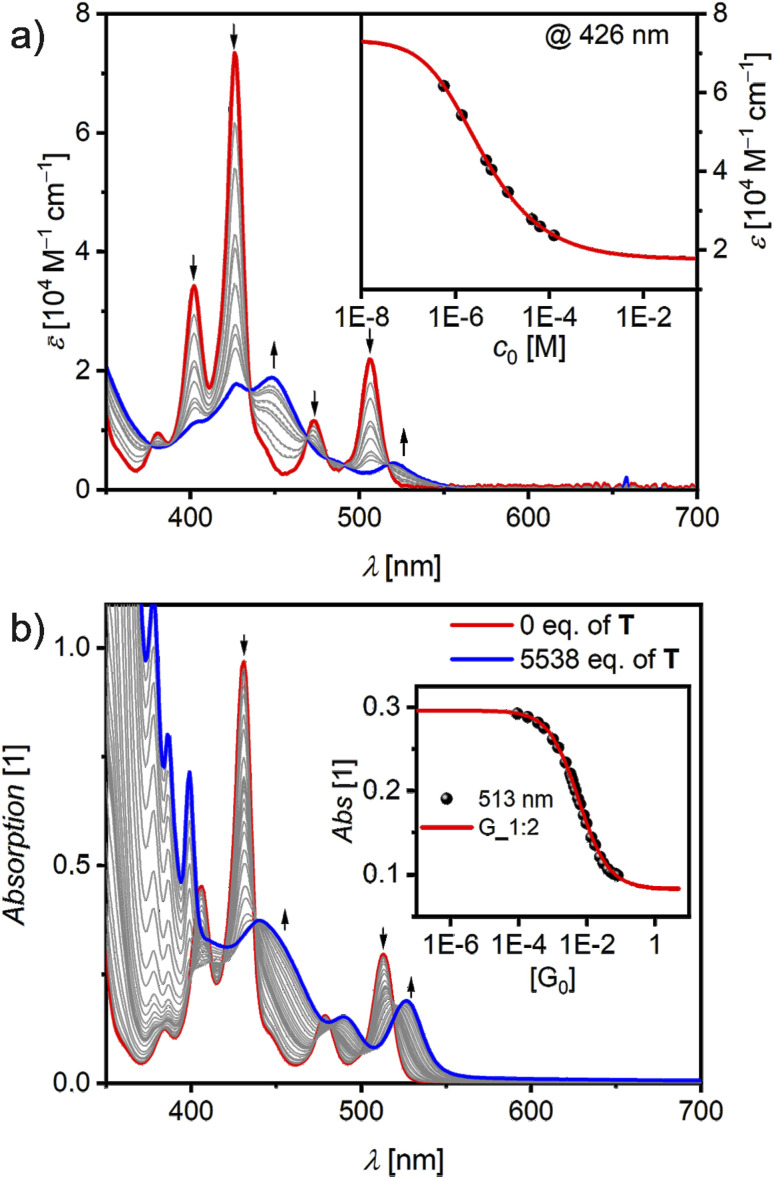
(a) Concentration-dependent UV/vis absorption spectra (grey solid lines) for CBI 1 in MCH/TCE (85/15; *c*_0_ = 1.26 × 10^−4^ – 5.66 × 10^−7^ M) at 298 K. The dashed (grey) and solid lines represent the calculated monomer (red) and dimer (blue) spectra according to a global fit analysis by the monomer–dimer model. The arrows indicate the spectral changes with increasing concentration. The inset depicts the monomer–dimer fit (red line) at *λ*_max_ = 426 nm (black symbols). (b) UV/vis absorption spectra (solid lines) for a solution of CBI 1 (*c*_0_ = 1.45 × 10^−5^ M, red line) and changes upon addition of T as guest (grey to blue lines, 5538 eq.) in TCE at 298 K. Arrows depict spectral changes with increasing amount of the guest T. Inset shows the absorption at *λ*_max_ = 513 nm (black symbol) with nonlinear curve to the 1 : 2 (red line) global (490–550 nm) model.

Different from other cocrystalline CT complexes,^16^ the large π-surface of CBI can provide sufficiently large Gibbs π–π-stacking energies with aromatic guest molecules that are mostly governed by dispersion forces, *i.e.* related to the area of the interacting surfaces.^41^ This allows us to support our investigations on the emission colour tuning by environmental engineering in solid state samples by the related defined complexes formed in solution upon addition of the respective guest molecules. Firstly, the aggregation of 1 was examined in a mixture of methylcyclohexane/1,1,2,2-tetrachloroethane (MCH/TCE = 85/15) at 298 K. The concentration(*c*_0_)-dependent study shown in Fig. 3a displays spectral changes upon increasing *c*_0_ with multiple isosbestic points. The spectra at higher *c*_0_ are less structured than that of the monomer and spread over a larger wavelength range. The intensity of the S_0_–S_2_ absorption decreases and a new *λ*_max_ at 448 nm appears. The S_0_–S_1_ absorption also decreases and shifts bathochromically. As multiple isosbestic points are observable at 435, 469 and 517 nm, only two different species are present in the studied concentration range, indicating a dimerization process. Therefore, the experimental data were fitted according to a global monomer–dimer model^42^ over the 350–600 nm spectral range. The monomer spectrum calculated from those data almost perfectly matches the experimental absorption spectrum at 10^−5^ M in more polar CHCl_3_ where monomers prevail (Fig. 1b). A degree of aggregation (*α*_agg_) up to 90% can be reached at the solubility limit of 1 × 10^−4^ M in the respective solvent mixture (Fig. 3a and S7, SI). The dimerization constant in MCH/TCE (85/15) at 298 K is remarkably high with *K* = 3.0 × 10^5^ M^−1^, significantly exceeding that of the smaller PBI chromophore with the same terphenyl imide substituents (4.3 × 10^4^ M^−1^) which was determined in pure MCH.^32^ This enhanced thermodynamic driving force for dimerization arises from the by 40% larger π-surface of CBI 1 compared to PBI, resulting in stronger intermolecular interactions with a Gibbs free binding energy of Δ*G* = −31.2 kJ mol^−1^. Although aggregation of CBI derivatives has been reported before,^43,44^ the focus of these earlier works were different and did not provide dimers of defined size with such exceptionally high aggregation constant of 10^5^ M^−1^. Regarding the photoluminescence properties, the transition from monomers to dimers goes along with significant changes in wavelength and lifetime. Thus, whilst the monomer of CBI 1 shows a green short-lived emission at 516 nm in CHCl_3_ (Fig. 1b), the dimer 1_2_ exhibits an orange excimer-like emission *λ*_em_ at 594 nm, which is less structured compared to the monomer and shows an increased lifetime (*τ*_PL_) of 19.9 ns (Fig. S8, SI).

As the thermodynamic driving force for self-assembly of CBI 1 is reduced in more polar solvents like pure TCE, and since the single-crystal structure showed that the imide substituents form a cavity, host–guest titrations were performed with electron-rich P, T and I_2_Cz as guest molecules at 298 K, which have different electron-donating strength (Fig. S9, SI). Complexation with 1 is demonstrated by significant changes in UV/vis absorption spectra upon addition of guest molecules (Fig. 3b and S10–S12, SI). As exemplified by the titration with T in Fig. 3b there is a strong decrease of both CBI absorption bands with a concomitant bathochromic shift as T concentration increases, corroborating the formation of a CT complex. An initial quasi-isosbestic point at around 517 nm disappears at higher T concentrations, which clearly indicates the sequential guest binding *via* the initial 1 : 1 to a final 1 : 2 complex with binding constants of *K*_1_ = 199 and *K*_2_ = 17 M^−1^. Titration with P likewise shows a decrease of the CBI absorption as well as the formation of a broad CT band from around 550–650 nm. The binding constants are the highest for P with *K*_1_ = 1008 and *K*_2_ = 235 M^−1^ (Table S7, SI). This result complies with our expectations, given that P has the largest and most shape-complementary π-surface of all guests and can therefore form the strongest interactions with 1. Accordingly, for the titration with the smallest guest I_2_Cz we observe the lowest binding constants of *K*_1_ = 126 and *K*_2_ = 42 M^−1^. Still, compared to our previous studies on complexation with shielded PBIs with the same *meta*-terphenyl substituents, the formation of 1 : 2 complexes in solution is strongly supported by the increased CBI π-surface size.^32^ As expected from the CT character seen in the absorption spectra, pronounced changes can also be observed in the emission spectra of the complexes compared to that of the parent CBI which are all red shifted and less structured (Fig. S13 and Table S8, SI). For the complex with P the emission maximum is shifted most strongly to 680 nm, indicative for an exciplex, with the tail extending into the NIR region.

### Solid state PL measurements

**Fig. 4:**
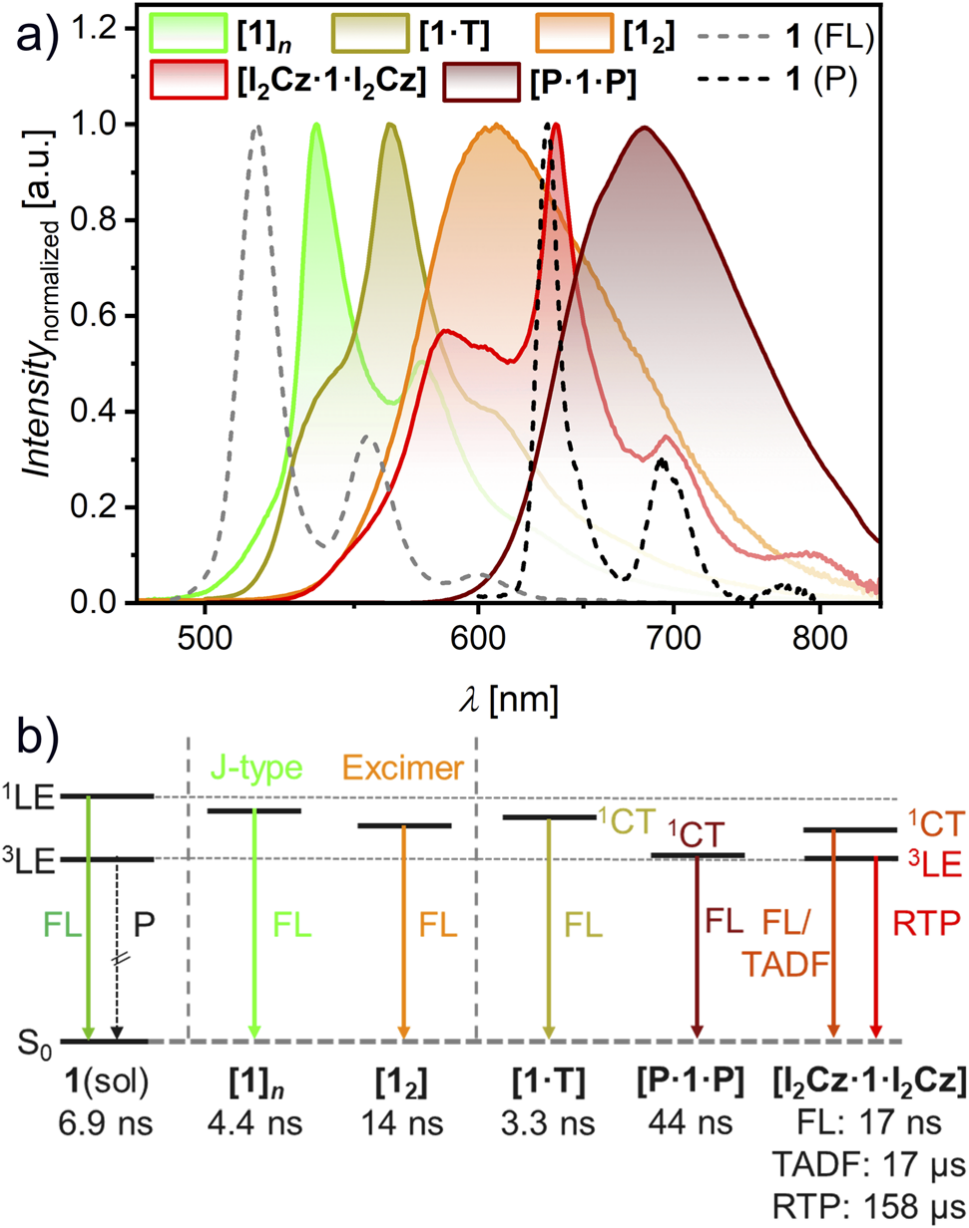
(a) PL spectra of respective microcrystals on Si/SiO_2_ substrate at 298 K upon UV irradiation ([1]*_n_*) or excitation at *λ*_ex_ = 440 nm ([1_2_]), 452 nm ([I_2_Cz·1·I_2_Cz]) or 476 nm ([1·T], [P·1·P]), respectively. Additionally, the fluorescence (FL, 298 K; dashed grey) and phosphorescence (P, 80 K; dashed black) of 1 in (frozen) CHCl_3_ solution are shown. (b) Schematic illustration of the energy levels of pristine 1 in solution and of the five (co)crystals. Additionally, the major lifetime components are given below.

As a most interesting part of this study, the emission properties of the five (co)crystals were investigated. Suitable crystallites were handpicked onto Si/SiO_2_ substrates (Fig. S14, SI) and ensembles of each were deposited on quartz substrates for the determination of *Φ*_PL_ (Fig. 4a and S15–S19, SI). The crystal [1]*_n_* with slip-stacked CBI molecules exhibits a well-resolved green emission with vibronic fine structure at an emission maximum (*λ*_em_) of 536 nm with a short lifetime (*τ*_PL_) of 4.44 ns and a *Φ*_PL_ of 9% (Fig. 4a, S20 and Table 1, SI). Our theoretical analysis, taking both Coulomb and CT coupling into account, suggests weak J-type coupling (*J*_total_ = −94 cm^−1^; Fig. S21 and Table S9, SI),^45,46^ which may contribute to the modest bathochromic shift of the emission band while retaining monomer-like vibronic structure, similar to that observed for the CBI 1 monomers in solution (Table 1).

**Table 1:** Summary of the optical properties of 1 as a monomer in solution as well as in the (co)crystalline state at 298 K. (Co)crystalline materials are marked by [guest·1·guest]

Material	*λ*_abs/CT_ [nm]	*λ*_em_ [nm]	*Φ*_PL_ b [%]	*τ*_PL_(Rel.)^c^ [ns(%)]	PL
1^a^ solution	430	516	31	6.86 (100)	^1^LE
[1]*_n_* crystal	440	536	9	4.44 (87)	^1^LE
				12.3 (13)	
[1_2_] crystal	472	608	19	14.0 (85)	EX
				28.3 (15)	
[1·T] crystal	474	563	16	3.30 (79)	CT
				8.87 (21)	
[I_2_Cz·1·I_2_Cz] crystal	468	587	<1	1.68 × 10^4^ (50)	TADF
		635		1.58 × 10^5^ (59)	RTP
[P·1·P] crystal	471	677	3	44.1 (100)	CT

a Measured in CHCl_3_.

b *Φ*_PL_ with absolute method at 298 K.

c *τ*_PL_ measured either with µs flash-lamp (TADF, RTP) or with ps laser (^1^LE, CT and EX) at RT and at their respective maxima with major lifetime components for mono- or biexponential fits.

The second single-crystal, composed of isolated dimers with substantial π–π-overlap, [1_2_] exhibits a bright and broad orange fluorescence (FL) at *λ*_em_ of 608 nm. This red-shifted and broadened emission, together with an increased *τ*_PL_ of 14.0 ns (with a second component of 28.3 ns) is consistent with excimer-like (EX) emission.^47^ This behavior is plausibly associated with stronger intermolecular interactions in the twisted, closely π-stacked dimer arrangement, which may facilitate enhanced coupling between transition dipole moments (*µ*_eg_) and allow for some degree of structural relaxation in the excited state.^48^ Calculations for the Coulomb and CT couplings for the dimer structure in the single crystal here show opposing values with a smaller negative, *i.e.* J-type, *J*_coul_ (−101 cm^−1^) and a larger positive, *i.e.* H-type, *J*_CT_ (573 cm^−1^; Fig. S21 and Table S9, SI). The weak Coulomb coupling for the CBI S_1_ state may not only arise from a non-ideal arrangement for J-type coupling but also a consequence of the rather small *µ*_eg_ of only 3.0 D. Therefore, CT (*J*_CT_) and vibronic couplings may be more dominant for determining the observable spectral changes.^45,46^ Still, this crystal [1_2_] has a high *Φ*_PL_ of 19% which like the spectral shape mimics the emission observed for the dimer aggregate in solution (Table 1; S8 and Fig. S8, SI).

The 1 : 1 cocrystal [1·T] has a yellow and structured emission with *λ*_em_ at 563 nm and *τ*_PL_ of 3.30 and 8.87 ns, indicating exciplex fluorescence emission with a *Φ*_PL_ of around 16%. Again, the shape of the emission spectrum is similar to that obtained for the 1 : 2 complex in solution as they originate from the same donor (T) and acceptor (1) pair (Table 1; S8 and Fig. S13, SI). The broad and structureless red emission of the 1 : 2 cocrystal [P·1·P], with a maximum at 677 nm and an increased lifetime of 44.1 ns is a clear case of exciplex fluorescence emission. Again, this emission mimics that of the complex formed by CBI 1 and P in solution after selective irradiation of the CT band (Table 1; S8 and Fig. S13, SI).

The most interesting case is the cocrystal [I_2_Cz·1·I_2_Cz] which exhibits a dual orange-red emission at room temperature, albeit with a *Φ*_PL_ of only 1% (Table 1). One emission band is broad with a maximum of *λ*_em_ at 587 nm, the other shows well-resolved vibronic progressions at a maximum of *λ*_em_ at 635 nm with *τ*_PL_ of 17 ns and 158 µs, respectively (Fig. S19, SI). The latter value indicates room-temperature phosphorescence (RTP) not only due to the long *τ*_PL_, but also to the fact that the structural shape and position match those of the phosphorescence emission from ^3^LE of 1 in solution sensitized by ethyl iodide at 80 K (Fig. 4a). The external heavy-atom effect (eHAE) of the iodine atoms attached to the carbazole guest can obviously effectively sensitize ISC.^19^ The lifetime measurement for the CT emission at 587 nm revealed both prompt fluorescence with *τ*_PL_ of 17.1 ns (Fig. S19c, SI) and a delayed component (see ungated and gated spectra in Fig. S19b, SI). By using a flash-lamp, a long lifetime from 16.8 up to 237 µs could be measured, indicating TADF emission. Therefore, ISC between the ^1^CT and the ^3^LE of CBI is present, as well as reverse ISC (RISC). These results demonstrate that the 1 : 2 complex exhibits dual TADF and RTP emission in the solid state. The prompt emission again matches the emission obtained in solution of the complex (Table 1; S8 and Fig. S13, SI).

Based on our experimental findings, we can now compare the energy levels of the emissive states for the (co)crystals with those of 1 in solution (Fig. 4b). The emissive states of the (co)crystals are all located below the locally excited singlet state (^1^LE) and higher than the triplet state (^3^LE) of the CBI chromophore. As expected from the energy levels of the frontier orbitals (Fig. S9, SI), the emission observed for the cocrystals becomes more red-shifted from T to I_2_Cz up to P. The ^1^CT state of [P·1·P] is here the lowest. For [I_2_Cz·1·I_2_Cz] the ^1^CT and ^3^LE states are energetically close which enables dual emission with TADF and RTP contributions. Notably, the ^3^LE state remains rather unchanged in the cocrystal as the position of the RTP proves. The anisotropy of all the cocrystals was also investigated using polarization-dependent microscopy (Fig. S22–S26, SI). All crystals showed a measurable dependence, with the strongest response observed for [1_2_] and [1·T], corresponding to an intensity reduction of about 70%.

### Temperature-dependent measurement of [I_2_Cz·1·I_2_Cz]

**Fig. 5:**
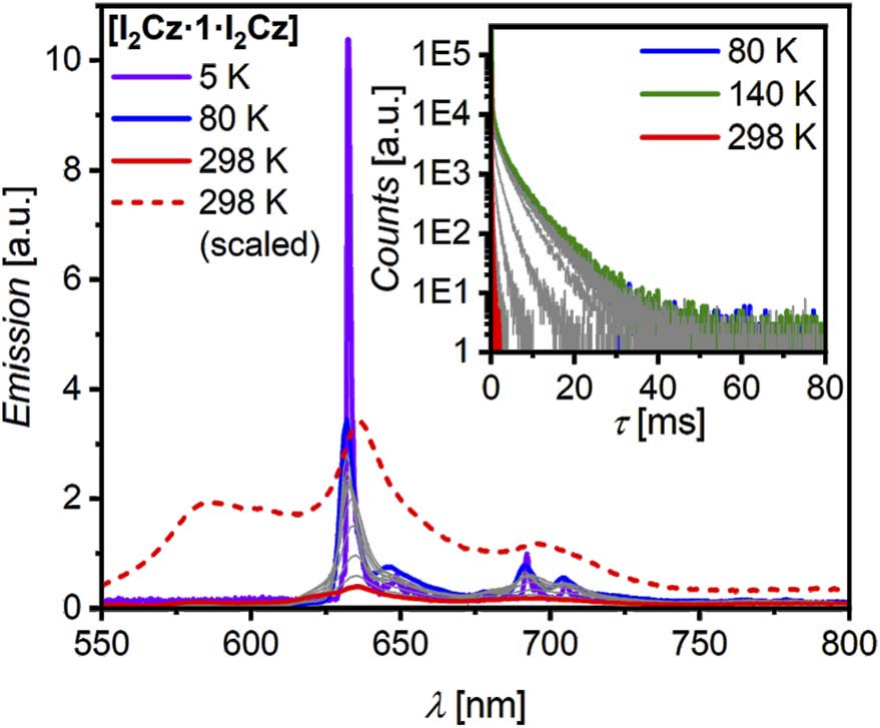
Temperature-dependent PL measurement of an ensemble of micro-cocrystals [I_2_Cz·1·I_2_Cz] under inert conditions on Si/SiO_2_ substrate upon excitation at *λ*_ex_ = 452 nm. PL spectra are displayed at 80 K (blue line), in steps of 20 K (grey lines) heating up to 298 K (red line). The spectrum at 5 K (*λ*_ex_ = 480 nm; solid violet line) and the scaled spectrum at 298 K (dashed red line) are also given. Inset shows the evolution of the PL lifetime starting from 80 K (blue line) upon heating in steps of 20 K (grey lines) up to 140 K (green line) and 298 K (red line).

To explore in more depth the emissive characteristics of the [I_2_Cz·1·I_2_Cz] cocrystal, temperature(*T*)-dependent experiments were performed under an inert argon atmosphere. Ensembles of cocrystalline samples deposited on Si/SiO_2_ substrates were cooled to 80 K, followed by stepwise heating (Δ*T* = 20 K) up to 298 K. Further, the PL spectrum at 5 K could be measured (Fig. 5). Upon cooling [I_2_Cz·1·I_2_Cz] down to 80 K a strong increase of the emission intensity and a more structured spectrum is observed which is explained by the suppression of thermal lattice vibrations and the deactivation of RISC towards TADF. Upon further cooling to 5 K the broad CT emission at 587 nm vanishes completely and *τ*_PL_ further increases to over 5 ms (80 K, 635 nm), while the spectral shape remains rather unchanged, resulting from a reduction in non-radiative decay. Increasing the temperature to room temperature causes the emission intensity and lifetime to decrease significantly (Table S10, SI). The *T*-dependent PL study verifies that [I_2_Cz·1·I_2_Cz] exhibits phosphorescence emission (Fig. S27, SI). To the best of our knowledge, this is the first pure organic CBI-based cocrystal showing RTP and TADF emission.

## Conclusions

With the attachment of two bulky *meta*-terphenyl substituents at the imide nitrogen, a sterically shielded coronene bisimide (CBI) chromophore could be synthesized. By this means, the formation of extended π-stacks could be prohibited and instead either dimer aggregates or 1 : 2 host-guest complexes could be obtained. Thus, self-assembly in low-polarity methylcyclohexane/1,1,2,2-tetrachloroethane (MCH/TCE) solvent mixtures afforded discrete dimeric π-stacks characterized by a high binding affinity of 3.0 × 10^5^ M^−1^ at 298 K. Further, the electron-poor CBI can act as an efficient host system for various electron-rich guest molecules as demonstrated for perylene (P), triphenylene (T), and 3,6-diiodocarbazole (I_2_Cz). Formation of 1 : 2 charge-transfer (CT)-complexes has been demonstrated for all three guests in TCE solution, with binding constants as high as *K*_1_ = 1008 M^−1^ and *K*_2_ = 235 M^−1^ for P. Five different single-crystal structures could be obtained that show a broadly tunable emission colour ranging from green up to the NIR. With almost identical and well-characterized complexes being accessible both in solution (due to the high binding affinity of the large CBI π-surface) and in solid state (due to the prohibition of more extended π-stack formation by the sterically demanding imide substituents), a unique investigation of emission properties became possible. Thus, two different crystals of the pristine CBI 1 showed very similar emission properties as observed for the monomer and the dimer aggregate in solution. One crystal exhibits an H-type dimer packing with orange excimer-type emission and the highest quantum yield of all the crystals. The other crystal exhibits a slipped-stacked packing arrangement which afforded a green monomer-like fluorescence and quantum yield of 9%. Next, by uptake of T and P in the receptor pockets of CBI co-crystals were obtained that show yellowish (16%) and red (3%) exciplex fluorescence, respectively. Most remarkably, the co-crystal of CBI 1 with I_2_Cz (1 : 4) exhibited a red-coloured dual emission originating from the ^1^CT state (and involving both prompt and delayed fluorescence *via* thermally activated delayed fluorescence (TADF)) and a long-lived room-temperature phosphorescence (RTP) from CBI's ^3^LE state. This work demonstrates that the emission of CBI systems can be tuned across a broad spectrum (from green to NIR) by controlling self-assembly and guest binding. This establishes a fundamental design principle, whereby the supramolecular organisation and host–guest interactions of a structurally defined CBI platform collectively determine the emission colour and excited-state dynamics.

## Author contributions

S. S.: conceptualization, investigation, formal analysis, visualization, writing – original draft; Ö. Ö.: investigation, visualization; K. S.: investigation (crystallography), formal analysis (crystallography); D. H.: investigation (spectroscopy), writing – review & editing; J. D.: investigation (spectroscopy); S. H.: resources; S. K.: resources, writing – review & editing; M. S.: conceptualization, investigation (spectroscopy), formal analysis (spectroscopy), supervision, writing – review & editing; F. W.: conceptualization, resources, supervision, writing – review & editing, founding acquisition.

## Conflicts of interest

There are no conflicts to declare.

## Supplementary Material

SC-017-D6SC02467J-s001

SC-017-D6SC02467J-s002

## Data Availability

CCDC 2540045 [1]*_n_*, 2540046 [1_2_], 2540047 [1·T], 2540048 [P·1·P] and 2540049 [I_2_Cz·1·I_2_Cz] contain the supplementary crystallographic data for this paper.^49*a–e*^ The data underlying this study are available in the supplementary information (SI) and in Zenodo, an open research repository, at https://doi.org/10.5281/zenodo.19203936. Supplementary information is available. See DOI: https://doi.org/10.1039/d6sc02467j.
