# Supplementary material for: Colour by design: tuning the solid-state emission of coronene bisimide by tailored matrices
Source: Chem Sci. 2026 May 28;17(27):13407–15. doi: 10.1039/d6sc02467j (PMC13234765; doi:10.1039/d6sc02467j)
Supplement: SC-017-D6SC02467J-s001 [file SC-017-D6SC02467J-s001.pdf]

Supplementary Information (SI) for Chemical Science.
This journal is © The Royal Society of Chemistry 2026

Supporting Information

Colour by Design: Tuning the Solid-State Emission of Coronene Bisimide by Tailored Matrices

Simon Soldner,[a] Ömer E. Öçal,[a] Kazutaka Shoyama,[a,b] Dominik Horneber,[c,d] Johannes Düreth,[c] Sven Höfling,[c] Sebastian Klembt[c,d] Matthias Stolte,[a,b] and Frank Würthner*[a,b]

[a] Center for Nanosystems Chemistry (CNC), Universität Würzburg, Theodor-Boveri-Weg, 97074 Würzburg, Germany.

[b] Institut für Organische Chemie, Universität Würzburg, Am Hubland, 97074 Würzburg, Germany.

[c] Julius-Maximilians-Universität Würzburg, Physikalisches Institut, and Würzburg-Dresden Cluster of Excellence ctd.qmat, Lehrstuhl für Technische Physik, Am Hubland, Würzburg, 97074, Germany.

[d] Julius-Maximilians-Universität Würzburg, Physikalisches Institut, and Würzburg-Dresden Cluster of Excellence ctd.qmat, Lehrstuhl für Experimentelle Physik 1, Am Hubland, Würzburg, 97074, Germany.

*E-mail: frank.wuerthner@uni-wuerzburg.de

Table of Contents

1. Materials and Methods S2
2. Synthesis S6
3. Characterization S10
4. Microscopic Pictures of Crystals S10
5. Single- and Cocrystal X-ray Analysis S11
6. Aggregation Study S23
7. Theoretical Calculation S24
8. Complexation Studies S25
9. X-Ray Diffraction Measurements S28
10. PL Measurement of Crystals S29
11. Calculations for Exciton Coupling in $[1]_n$ and $[1_2]$ Crystals S33
12. Polarization-dependence of Single- and Cocrystals S34
13. Temperature-dependent PL Measurement S37
14. NMR Spectroscopy S39
15. Mass Spectrometry S42
16. References S44

1. Materials and Methods

All chemicals, reagents and solvents were purchased from commercial suppliers and used after appropriate purification if not stated otherwise. All reactions were done under inert conditions. The CBI **1** was synthesized according to an optimized literature known procedure for imidization of perylene tetraester.[S1,S2] The precursors were also synthesized according to literature known procedures.[S3,S4,S5] Dichloromethane (DCM) was distilled prior to use. Column chromatography was performed using commercial glass columns packed with silica gel 60 M (particle size of 0.04–0.063 mm from Merck KGaA) stationary phase. Normal phase high-performance liquid chromatography (HPLC) was performed on a JASCO recycling semipreparative HPLC system equipped with a VP 250/21 NUCLEOSIL 100-7 column from Macherey-Nagel. Perylene (**P**; ≥95%) was purchased from BLD pharm and recrystallized in DCM/methanol. Triphenylene (**T**; ≥98%) and 3,6-Diiodocarzabzole (**I_2Cz**; ≥99.82%) were purchased from Sigma-Aldrich and BLD pharm, respectively, and used without further purification.

Crystallization methods. The single crystals of CBI **1** were grown by slow diffusion of methanol into a 10^{-3} M solution of chloroform ($CHCl_3$) or iodobenzene (IBz). The same method was used to grow the cocrystals of CBI **1** combined with **P**, **T** and **I_2Cz**. Also, methanol (**P** and **T**) or *n*-hexane (**I_2Cz**) slowly diffused into 10^{-3} M solution of toluene (**P** and **T**) or $CHCl_3$ (**I_2Cz**). The ratio between bisimide and guest varied: CBI:**P** = 1:2, CBI:**T** = 1:2 and CBI:**I_2Cz** = 1:4.

UV/Vis spectroscopy was carried out at 298 K on a JASCO V770 spectrophotometer equipped with a Peltier system. Solution spectra were measured using 1 mm, 10 mm and 100 mm cuvettes (SUPRASIL®, Hellma® Analytics) and the solvents chloroform ($CHCl_3$), methylcyclohexane (MCH) and 1,1,2,2-tetrachloroethane (TCE) were spectroscopic grade. For concentration-dependent UV/Vis absorption studies, stock solutions were stepwise diluted before each measurement.

Complexation studies in solution. UV/Vis absorption titration experiments were conducted by applying the constant-host routine in TCE solution. For this purpose, a solution of host (c_0(**1**) = 10^{-5} M) with a defined excess of guest was titrated to a pure host solution with the same CBI concentration. The obtained data were fitted globally with a 1:2 binding model by a nonlinear regression method using the program bindfit[S6] from Thordarson and colleagues.[S7]

Photoluminescence spectroscopy was carried out on a FLS980-D2D2-ST spectrometer from Edinburgh Instruments. Spectra were corrected against the photomultiplier sensitivity and the lamp intensity. Crystalline samples on quartz or Si/SiO_2 substrates were investigated with a front-face sample holder F-J03 in 22.5° geometry. Temperature-dependent photoluminescence spectra (80−298 K) were recorded using an Oxford Instruments nitrogen flow cryostat connected to an Oxford ITC601 temperature controller. Fluorescence lifetimes were determined with EPL picosecond pulsed diode laser (λ_{ex} = 479.7 nm) for time correlated single photon counting (TCSPC) with an Edinburgh Instruments FLS980-D2D2-ST spectrometer. Phosphorescence lifetimes were determined with a Xe-Flash400 lamp. The absolute photoluminescence quantum yield (Φ_{PL}) of **1** in solution and the cocrystal powders were determined on a Hamamatsu Absolute PL Quantum Yield Measurement System CC9920-02. The system is composed of a 150 W CW Xenon lamp as the excitation source, a monochromator (250−700 nm, full width at half-maximum (*FWHM* = 10 nm), an integrating sphere, and a multichannel spectrometer capable of simultaneously measuring multiple wavelengths between 300 nm and 900 nm. Φ_{PL} were determined using the absolute method without correction for reabsorption for crystals and with correction in solution.[S8]

NMR spectroscopy was performed using Bruker Avance DMX 600 MHz and Bruker Avance III HD 400 MHz NMR spectrometers. Chemical shifts (δ) are listed in parts per million (ppm) relative to residual undeuterated solvent signals. The multiplicities for proton signals are abbreviated as s, d, t and m for singlet, doublet, triplet and multiplet, respectively.

High resolution mass spectrometry. Matrix-assisted laser desorption/ionization – time of flight (MALDI-TOF) measurements were performed on a Bruker Daltonics ultrafleXtreme mass spectrometer. *trans*-2-[3-(4-*tert*-butylphenyl)-2-methyl-2-propenylidene]-malononitrile (DCTB) was used as the matrix.

Melting points were determined using an SMP50 from Stuart Equipment.

Single crystal X-ray diffraction. Measurements were performed at 100 K on a Bruker D8 Quest Kappa diffractometer with a Photon II CPAD as detector (**[I_2Cz·1·I_2Cz]**). In addition, measurements were performed at DESY (application numbers I-20230262 and I-20231007) with the P11 beamline at 100 K, using a single 360° scan ϕ (**[1]**$_n$, **[1_2]**, **[T·1]** and **[P·1·P]**). The diffraction data were processed using the XDS software package.[S9] The diffraction data collected by Bruker diffractometer were processed with the help of the APEX3 and APEX4 program packages. The structure was solved with the help of the SHELXT[S10] software and

subsequently further processed using Fourier techniques, where SHELXL[S11] software was used. Structure verification by PLATON[S12,S13] showed level A alerts for **[1]*n*** and **[1₂]**, which were caused by heavy disorder of solvent or side chains, and overall low crystallinity of these crystals. These issues are addressed in the later sections along with the corresponding structure data. Crystallographic data are deposited on the Cambridge Crystallographic Data Centre as supplementary publication numbers 2540045, 2540046, 2540047, 2540048 and 2540049 for **[1]*n***, **[$\mathbf{1_2}$]**, **[1·T]**, **[P·1·P]** and **[$\mathbf{I_2}$Cz·1·$\mathbf{I_2}$Cz]**, respectively.

Wide angle X-ray scattering (WAXS) measurements were carried out on a D8 Quest diffractometer equipped with a 2D Photon II 14 CPAD detector (both from Bruker AXS GmbH). The detector was positioned at a distance of 10 cm with a detection angle of 0° (WAXS pattern in white/black). WAXS data were processed and evaluated with the X-ray analysis program Datasqueeze (version 3.0.23) with calibration to AgBh.[S14-S16]

(Photoluminescence) polarization microscopy and spectroscopy of single crystals. (Photoluminescence, PL) Polarization microscopy images of micrometre-sized single crystals on quartz substrates were recorded with a Zeiss Axio Imager optical polarization microscope. Localized PL spectra of individual single crystals were measured with the same microscope with a customized spot resolution down to 1 × 1 μm^2 (A. S. & Co GmbH) coupled to an Ocean Optics Maya 2000Pro® spectrometer.

Theoretical calculations. Frontier molecular orbital energies were calculated from **1**, **T**, **$\mathbf{I_2}$Cz** and **P** structures optimized at the r^2SCAN-3c/def2-mTZVP level with ORCA 6.1.1.[S 17] Coulomb coupling (J_{Coul}) was calculated with TD-DFT at the ωB97/def2-SVP level with Gaussian09,[S18] taking the average of J_{Coul} for the first two allowed states (**[$\mathbf{1_2}$]**) and J_{Coul} for the allowed second state (**[1]*n***). Charge transfer coupling (J_{CT}) was calculated with the energy splitting in the dimer model at the B3LYP/def2-SVP level with ORCA 6.1.1.[S17] All calculations were carried out on the **[$\mathbf{1_2}$]** and **[1]*n*** crystal structures.

Temperature-dependent PL measurement. For photoluminescence (PL) measurements conducted at temperatures below 80 K, a helium flow cryostat (Janis ST-500) in combination with a Fourier spectroscopy setup was employed. Excitation was provided by a supercontinuum white-light source (NKT Super-K Fianium) spectrally filtered to 480 nm. The excitation beam was focused onto the sample surface using a high-numerical-aperture objective (NA = 0.65, Mitutoyo M Plan Apo NIR HR 50×), yielding a laser spot with an approximate diameter of 12 μm. The emitted PL signal was collected in reflection geometry through the same objective,

spectrally filtered using a 500 nm long-pass filter, and subsequently guided to the entrance slit of a 500 mm Czerny–Turner spectrometer (Andor Shamrock 500i). Signal detection was performed using a Peltier-cooled EMCCD camera (Andor Newton 971).

2. Synthesis

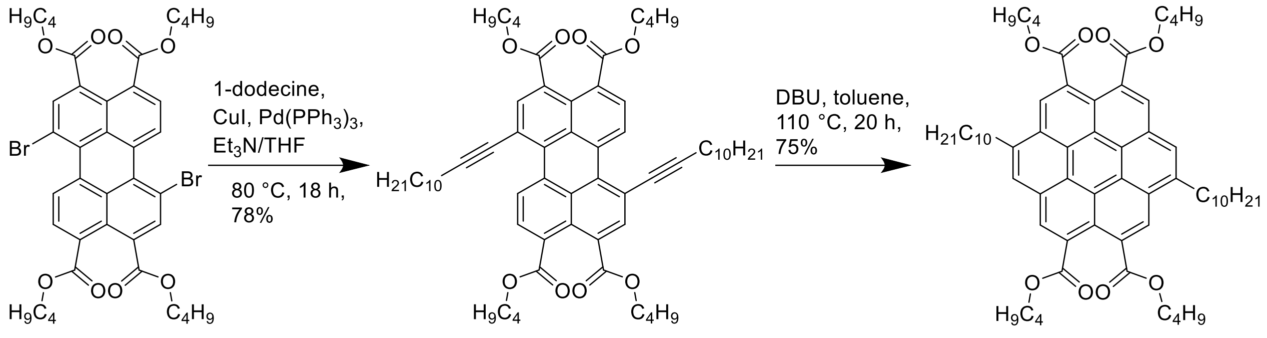

4 **5** **2**

Scheme S1. Synthesis of the precursor **2**.

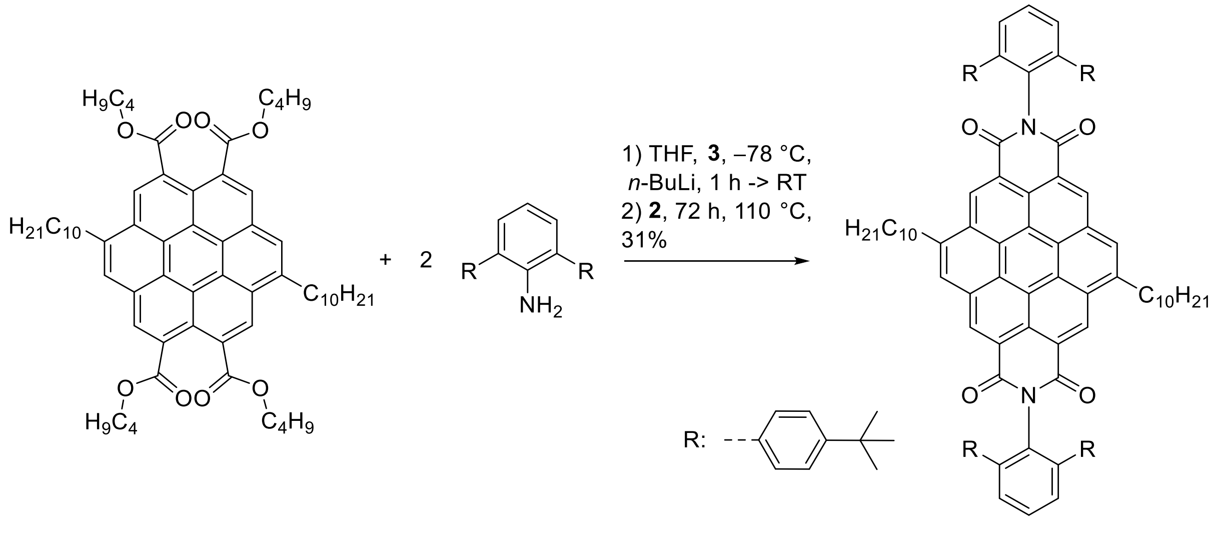

2 **3** **1**

Scheme S2. Synthesis of CBI **1**.

Synthesis of 1,7-di-1-dodecinperylene-3,4:9,10- tetracarboxy tetrabutylester (5):

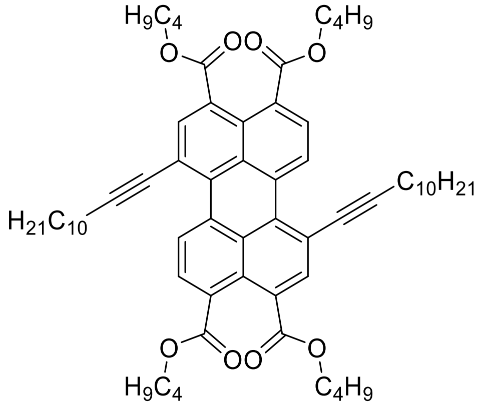

5

1,7-Dibromoperylene-3,4,9,10-tetracarboxy tetrabutylester (**4**) (1.29 g, 1.59 mmol, 1.0 eq.) was added to a mixture of THF (80 mL) and triethylamine (80 mL), which was degassed by the freeze-pump-thaw technique twice. Then copper iodide (30.2 mg, 159 µmol, 10 mol %), 1-dodecyne (1.36 mL, 1.06 g, 6.35 mmol, 4.0 eq.) and tetrakis(triphenylphosphine)palladium(0) (183 mg, 159 µmol, 10 mol %) were added and the mixture was degassed once more. The reaction mixture was stirred for 18 h at 80 °C. After cooling the mixture to room temperature, the solvents were removed under reduced pressure. Then DCM (50 mL) was added, washed with water (2 × 50 mL) and dried over magnesium sulfate. After removing the solvent under reduced pressure, the crude product was purified by column chromatography (silica-gel, cyclohexane/DCM = 5/1).

Yield: 1.22 g (1.24 mmol, 78%) of an orange oily substance.

^{1}H-NMR (400 MHz, $CDCl_3$, 295 K): δ/ppm = 9.51−9.49 (d, J = 8.1 Hz, 2 H), 8.14 (s, 2 H), 8.05−8.03 (d, J = 8.1 Hz, 2 H), 4.34−4.31 (m, 8 H), 2.56−2.53 (t, J = 7.4 Hz, 4 H) 1.81−1.74 (m, 8 H), 1.73−1.66 (m, 4 H), 1.53−1.44 (m, 12 H), 1.38−1.27 (m, 24 H), 1.01−0.96 (m, 12 H), 0.88−0.85 (t, J = 7.1 Hz, 6 H).

^{13}C-NMR (101 MHz, $CDCl_3$, 295 K): δ/ppm = 168.4, 168.0, 136.4, 133.2, 132.4, 130.3, 130.1, 129.4, 129.3, 126.8, 126.3, 118.9, 97.7, 82.0, 65.4, 65.3, 31.9, 30.6, 29.6, 29.5, 29.3, 29.2, 28.4, 22.7, 20.1, 19.2, 19.2, 14.1, 13.8.

HRMS (MALDI-TOF, pos. Mode, DCM/DCTB): m/z calcd. for $C_{64}H_{84}O_8$: 980.61662; found: 980.63835.

Synthesis of 3,9-didecylcoronene-1,12:6,7-tetracarboxy tetrabutylester (2):

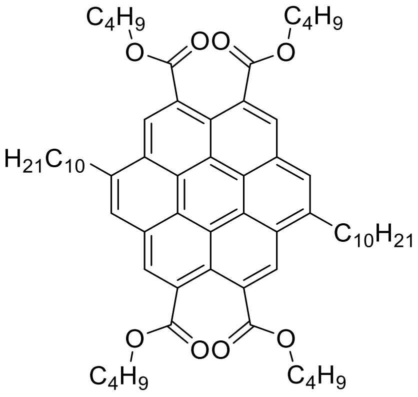

2

1,7-Di-1-dodecinperylene-3,4,9,10-tetracarboxylic acid tetrabutyl ester (**5**) (473 mg, 316 µmol, 1.0 eq.) was dissolved in toluene (76 mL). The mixture was degassed three times by the freeze-pump-thaw technique. After adding DBU (45.0 mg, 44.1 µL, 296 µmol, 58 mol %), the mixture was stirred for 20 h at 120 °C. After cooling the mixture to room temperature, the solvent was removed, the resulting solid was purified by column chromatography (silica-gel, cyclohexane/DCM = 1/2) and then recrystallized from DCM/acetone.

Yield: 336 mg (224 µmol, 71%) of a yellow-greenish solid.

^{1}H-NMR (400 MHz, $CDCl_3$, 295 K): δ/ppm = 9.57 (s, 2 H), 9.35 (s, 2 H), 8.82 (s, 2 H), 4.59−4.55 (t, J = 7.78 Hz, 8 H), 3.80−3.76 (t, J = 7.84 Hz, 4 H), 2.19−2.11 (m ,4 H), 1.96−1.88 (m, 8 H), 1.68−1.56 (m, 12 H), 1.38−1.26 (m, 24 H), 1.07-1.03 (m, 12 H), 0.89−0.85 (t, J = 6.39 Hz, 6 H).

^{13}C-NMR (101 MHz, $CDCl_3$, 295 K): δ/ppm = 169.6, 169.4, 139.8, 129.9, 128.9, 128.8, 128.6, 127.8, 127.7, 125.8, 124.4, 123.7, 122.4, 121.7, 65.7, 65.6, 33.9, 31.9, 31.4, 30.8, 30.8, 30.0, 29.7, 29.7, 29.6, 29.3, 22.7, 19.4, 19.4, 14.1, 13.9, 13.9.

HRMS (MALDI-TOF, pos. Mode, DCM/DCTB): m/z calcd. for $C_{64}H_{84}O_8$: 980.61662; found: 980.68614.

Melting Point: 131 °C.

Synthesis of *N*,*N′*-bis-[2,6-bis(4-*tert*-butylphenyl)]-3,9-didecylcoronene-1,12:6,7-tetracarboxybisimide (1):

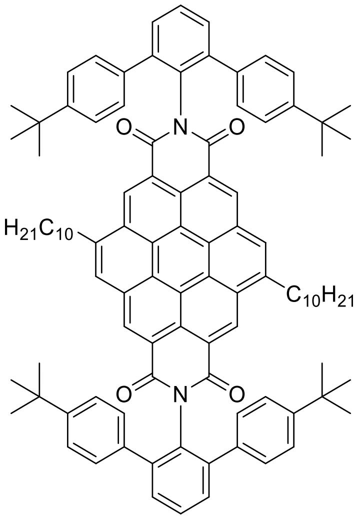

1

2,6-Bis(4-*tert*-butylphenyl)aniline (**3**) (30.9 mg, 86.5 µmol, 4.2 eq.) was dissolved in THF (1.0 mL) under nitrogen atmosphere in a pressure-stable Schlenk-tube. The mixture was cooled down to −78 °C. Then a solution of *n*-BuLi (2.5 M, 69.2 µL, 173 µmol, 8.5 eq.) in *n*-hexane was added. The mixture was stirred for 1 h at this temperature. After heating to room temperature, **2** (20.0 mg, 20.4 µmol, 1.0 eq.) was added and heated at 110 °C for 72 h. After cooling to room temperature, the crude product was concentrated under vacuum and purified by column chromatography (silica-gel, dichloromethane) and by HPLC (dichloromethane).

Yield: 8.90 mg (6.36 µmol, 31%) of an orange solid.

^{1}**H-NMR** (600 MHz, $C_2D_2Cl_4$, 374 K): δ/ppm = 9.88 (s, 2 H), 9.66 (s, 2 H), 8.90 (s, 2 H), 7.63−7.60 (t, J = 7.64 Hz, 2 H), 7.55−7.54 (d, J = 7.84 Hz, 4 H), 7.38−7.37 (d, J = 8.50 Hz, 8 H), 6.98−6.97 (d, J = 8.71 Hz, 8 H), 3.80−3.77 (t, J = 8.43 Hz, 4 H), 2.12−2.10 (m, 4 H), 1.65-1.63 (m, 4 H), 1.28-1.26 (m, 24 H), 0.85−0.84 (m, 6 H), 0.80 (s, 36 H).

HRMS (MALDI-TOF, pos. Mode, DCM/DCTB): *m*/*z* calcd. for $C_{100}H_{106}N_2O_4$: 1398.81526; found: 1398.90876.

Melting Point: >350 °C.

UV/Vis ($CHCl_3$; 1.2 × 10^{-5} M): λ_{max}/nm (ε/$M^{-1}cm^{-1}$) = 430 (71800).

Fluorescence ($CHCl_3$): λ_{max}/nm = 516.

3. Characterization

Table S1. Optical properties of CBI **1** in $CHCl_3$ at room temperature.

Substance	λ_{abs} (S_0-S_1) [nm]	λ_{abs} (S_0-S_2) [nm]	ε (S_0-S_1) [$M^{-1}\,cm^{-1}$]	ε (S_0-S_2) [$M^{-1}\,cm^{-1}$]	λ_{em} [nm]	τ_{PL} [ns]	ϕ_{PL} [a] [%]
**1**	512	430	21100	71800	516	6.86	31

[a] Quantum yield was determined with an integrating sphere setup and is corrected for reabsorption.

4. Microscopic Pictures of Crystals

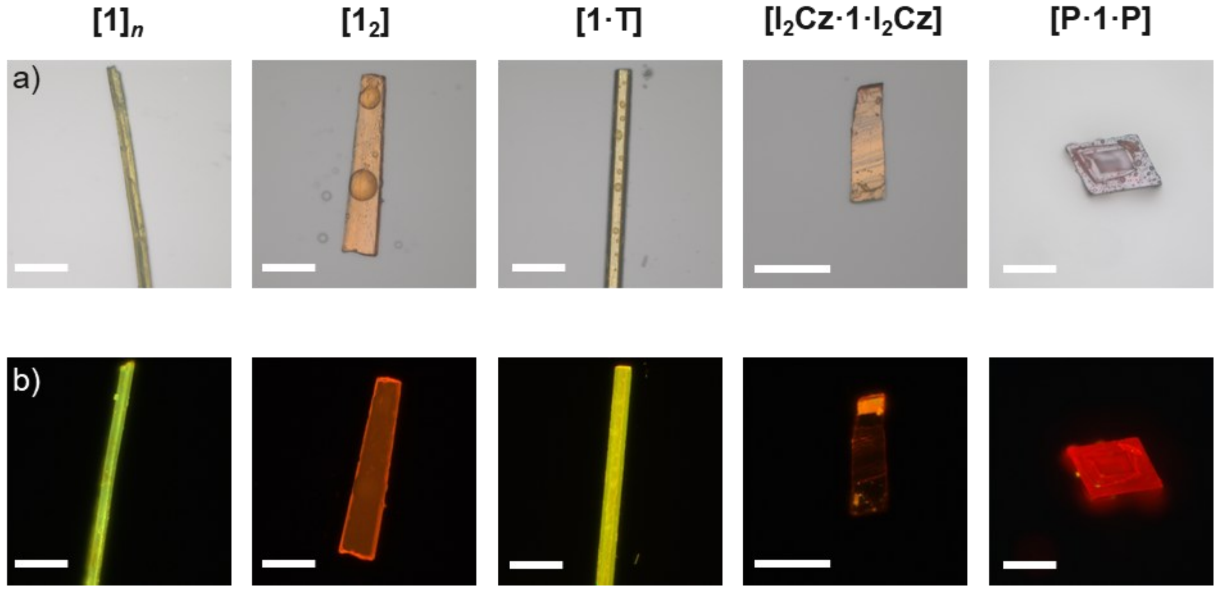

Figure S1. Microscopic pictures of the two crystals of neat CBI **1** as well as cocrystals with **T**, $\mathbf{I_2Cz}$ and **P** on quartz substrates (left to right). The pictures show crystals under a) ambient light as well as b) upon UV irradiation. The scale bars equal each 100 µm.

5. Single- and Cocrystal X-ray Analysis

Table S2. Crystallographic data and structure refinements for CBI **1** single crystal **[1]*ₙ***.

Identification code	CCDC 2540045	
Empirical formula	$C_{103.19}H_{106}N_2O_{7.19}$	
Formula weight	1489.25	
Temperature	100(2) K	
Wavelength	0.61991 Å	
Crystal system	Triclinic	
Space group	$P\bar{1}$	
Unit cell dimensions	a = 6.824(4) Å	α = 78.600(12)°.
	b = 17.592(6) Å	β = 89.465(19)°.
	c = 35.792(3) Å	γ = 88.533(18)°.
Volume	4211(3) $Å^3$	
Z	2	
Density (calculated)	1.175 g/cm^3	
Absorption coefficient	0.055 mm^{-1}	
F(000)	1593.3	
Crystal size	0.050 × 0.020 × 0.010 mm^3	
Theta range for data collection	0.506 to 28.313°.	
Index ranges	$-8 \leq h \leq 8$, $-22 \leq k \leq 22$, $-48 \leq l \leq 48$	
Reflections collected	23001	
Independent reflections	23001 [R_{int} = 0.2002]	
Completeness to θ = 21.836°	97.2%	
Refinement method	Full-matrix least-squares on F^2	
Data / restraints / parameters	23001 / 2093 / 1238	
Goodness-of-fit on F^2	1.066	
Final R indices [$I > 2\sigma(I)$]	R_1 = 0.1454, wR_2 = 0.3820	
R indices (all data)	R_1 = 0.3342, wR_2 = 0.4914	
Extinction coefficient	n/a	
Largest diff. peak and hole	0.344 and −0.421 e·$Å^{-3}$	

Structure verification for [1]$_n$

PLAT026

PROBLEM: Ratio Observed / Unique Reflections (too) Low .. 24% Check

RESPONSE: The low ratio of observed to unique reflections is a consequence of the very large molecular structure and resulting large unit cell, combined with the low crystallinity. The crystal diffracted only weakly, especially at higher angles.

PLAT084

PROBLEM: High wR2 Value (i.e. > 0.25) .... 0.49 Report

RESPONSE: The high wR2 value is caused by heavy disorder in the solvent accessible voids and flexible side chains, combined with weak diffraction originating from the large unit cell and low crystallinity.

Table S3. Crystallographic data and structure refinements for CBI **1** single crystal **[1_2]**.

Identification code	CCDC 2540046	
Empirical formula	$C_{100}H_{106}N_2O_4$	
Formula weight	1399.87	
Temperature	100(2) K	
Wavelength	0.61989 Å	
Crystal system	Triclinic	
Space group	$P\bar{1}$	
Unit cell dimensions	a = 12.751(11) Å	α = 79.76(5)°.
	b = 20.811(17) Å	β = 83.11(2)°.
	c = 31.27(2) Å	γ = 79.64(2)°.
Volume	8000(11) $Å^3$	
Z	4	
Density (calculated)	1.162 g/cm^3	
Absorption coefficient	0.053 mm^{-1}	
F(000)	3008	
Crystal size	0.100 × 0.100 × 0.020 mm^3	
Theta range for data collection	0.579 to 23.154°.	
Index ranges	$-15 \leq h \leq 16$, $-26 \leq k \leq 26$, $-39 \leq l \leq 39$	
Reflections collected	33209	
Independent reflections	33209 [R_{int} = 0.1194]	
Completeness to θ = 21.835°	98.6%	
Absorption correction	None	
Refinement method	Full-matrix least-squares on F^2	
Data / restraints / parameters	33209 / 4076 / 1910	
Goodness-of-fit on F^2	1.382	
Final R indices [$I > 2\sigma(I)$]	R_1 = 0.2166, wR_2 = 0.5555	
R indices (all data)	R_1 = 0.3491, wR_2 = 0.6259	
Extinction coefficient	n/a	
Largest diff. peak and hole	0.515 and –0.367 e·$Å^{-3}$	

Structure verification for [1_2]

PLAT026

PROBLEM: Ratio Observed / Unique Reflections (too) Low .. 28% Check

RESPONSE: The low ratio of observed to unique reflections is a consequence of the very large molecular structure and resulting large unit cell, combined with the low crystallinity. The crystal diffracted only weakly, especially at higher angles.

PLAT082

PROBLEM: High R1 Value .... 0.22 Report

RESPONSE: The high R1 value is caused by heavy disorder in the flexible side chains, combined with weak diffraction originating from the large unit cell and low crystallinity.

PLAT084

PROBLEM: High wR2 Value (i.e. > 0.25) .... 0.63 Report

RESPONSE: The high wR2 value is caused by heavy disorder in the flexible side chains, combined with weak diffraction originating from the large unit cell and low crystallinity.

Table S4. Crystallographic data and structure refinements for cocrystal **[1·T]**.

Identification code	CCDC 2540047	
Empirical formula	$C_{271}H_{276}N_4O_8$	
Formula weight	3716.97	
Temperature	100(2) K	
Wavelength	0.61989 Å	
Crystal system	Monoclinic	
Space group	$P2_1/c$	
Unit cell dimensions	a = 11.982(4) Å	α = 90°.
	b = 28.663(4) Å	β = 90.52(6)°.
	c = 30.843(4) Å	γ = 90°.
Volume	10592(4) Å^3	
Z	2	
Density (calculated)	1.165 g/cm^3	
Absorption coefficient	0.053 mm^{-1}	
F(000)	3988	
Crystal size	0.100 × 0.030 × 0.010 mm^3	
Theta range for data collection	0.846 to 27.942°.	
Index ranges	$-18 \leq h \leq 18$, $-39 \leq k \leq 40$, $0 \leq l \leq 39$	
Reflections collected	30236	
Independent reflections	30236 [R_{int} = 0.0611]	
Completeness to θ = 21.835°	98.8%	
Absorption correction	None	
Refinement method	Full-matrix least-squares on F^2	
Data / restraints / parameters	30236 / 1207 / 1591	
Goodness-of-fit on F^2	1.052	
Final R indices [$I > 2\sigma(I)$]	R_1 = 0.1245, wR_2 = 0.3984	
R indices (all data)	R_1 = 0.1805, wR_2 = 0.4539	
Extinction coefficient	n/a	
Largest diff. peak and hole	0.589 and −0.515 e·Å^{-3}	

Table S5. Crystallographic data and structure refinements for cocrystal **[P·1·P]**.

Identification code	CCDC 2540048	
Empirical formula	$C_{140}H_{130}N_2O_4$	
Formula weight	1904.46	
Temperature	100(2) K	
Wavelength	0.61989 Å	
Crystal system	Monoclinic	
Space group	$P2_1/c$	
Unit cell dimensions	a = 15.447(13) Å	α = 90°.
	b = 22.601(5) Å	β = 108.390(9)°.
	c = 15.846(5) Å	γ = 90°.
Volume	5250(5) Å^3	
Z	2	
Density (calculated)	1.205 g/cm^3	
Absorption coefficient	0.055 mm^{-1}	
F(000)	2032	
Crystal size	0.100 × 0.100 × 0.020 mm^3	
Theta range for data collection	1.419 to 27.996°.	
Index ranges	$-20 \leq h \leq 20$, $-33 \leq k \leq 33$, $-22 \leq l \leq 22$	
Reflections collected	99296	
Independent reflections	15165 [R_{int} = 0.0532]	
Completeness to θ = 21.835°	98.8%	
Absorption correction	None	
Refinement method	Full-matrix least-squares on F^2	
Data / restraints / parameters	15165 / 321 / 797	
Goodness-of-fit on F^2	1.015	
Final R indices [$I > 2\sigma(I)$]	R_1 = 0.1298, wR_2 = 0.3743	
R indices (all data)	R_1 = 0.1882, wR_2 = 0.4422	
Extinction coefficient	n/a	
Largest diff. peak and hole	0.541 and –0.294 e·Å^{-3}	

Table S6. Crystallographic data and structure refinements for cocrystal **[I_2Cz·1·I_2Cz]**.

Identification code	CCDC 2540049	
Empirical formula	$C_{150}H_{136}Cl_6I_8N_6O_4$	
Formula weight	3314.54	
Temperature	100(2) K	
Wavelength	1.54178 Å	
Crystal system	Triclinic	
Space group	$P\bar{1}$	
Unit cell dimensions	a = 11.9797(14) Å	α = 77.575(6)°.
	b = 15.8387(17) Å	β = 78.866(10)°.
	c = 19.038(2) Å	γ = 89.072(4)°.
Volume	3460.1(7) Å^3	
Z	1	
Density (calculated)	1.591 g/cm^3	
Absorption coefficient	15.574 mm^{-1}	
F(000)	1636	
Crystal size	0.115 × 0.067 × 0.034 mm^3	
Theta range for data collection	2.423 to 72.289°.	
Index ranges	$-14 \leq h \leq 14$, $-19 \leq k \leq 19$, $-23 \leq l \leq 23$	
Reflections collected	87563	
Independent reflections	13630 [R_{int} = 0.0380]	
Completeness to θ = 67.679°	100.0%	
Refinement method	Full-matrix least-squares on F^2	
Data / restraints / parameters	13630 / 114 / 828	
Goodness-of-fit on F^2	1.022	
Final R indices [$I > 2\sigma(I)$]	R_1 = 0.0294, wR_2 = 0.0749	
R indices (all data)	R_1 = 0.0329, wR_2 = 0.0776	
Extinction coefficient	n/a	
Largest diff. peak and hole	1.664 and –0.710 e·Å^{-3}	

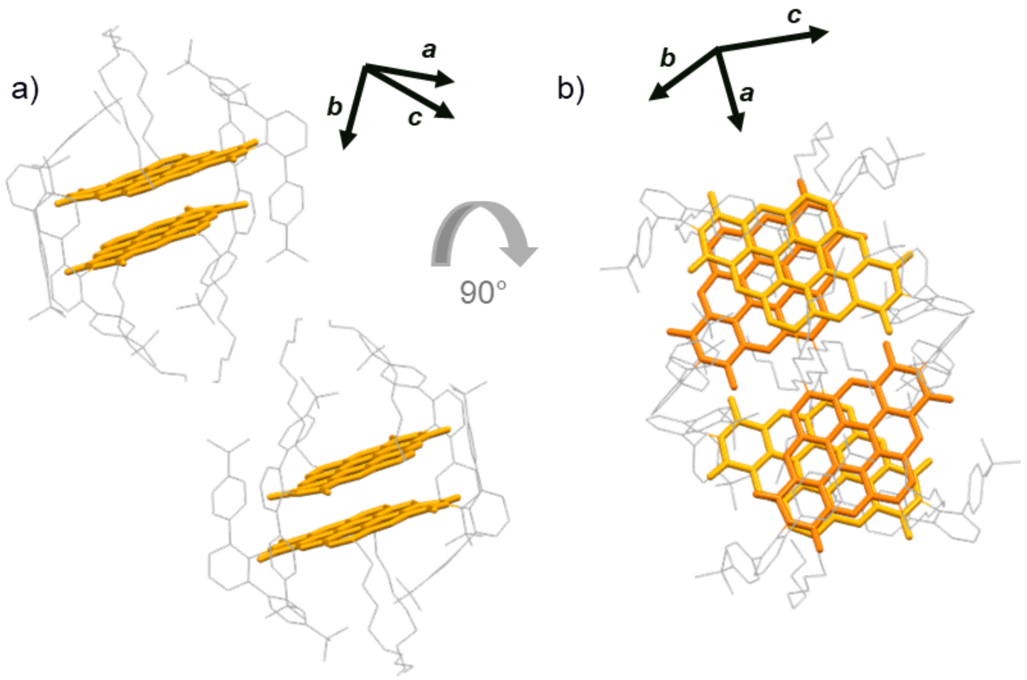

Figure S2. Packing of CBI **1** as dimeric π-stacks **[1$_2$]** in the single crystal grown from $CHCl_3$ solution (c_0 = 10^{-3} M) by slow diffusion of methanol according to X-ray analysis shown in a) side- and b) top-view. The CBI chromophore (orange) is displayed in capped sticks, whereas the imide substituents and the substituents at the CBI core are displayed in wireframe (grey).

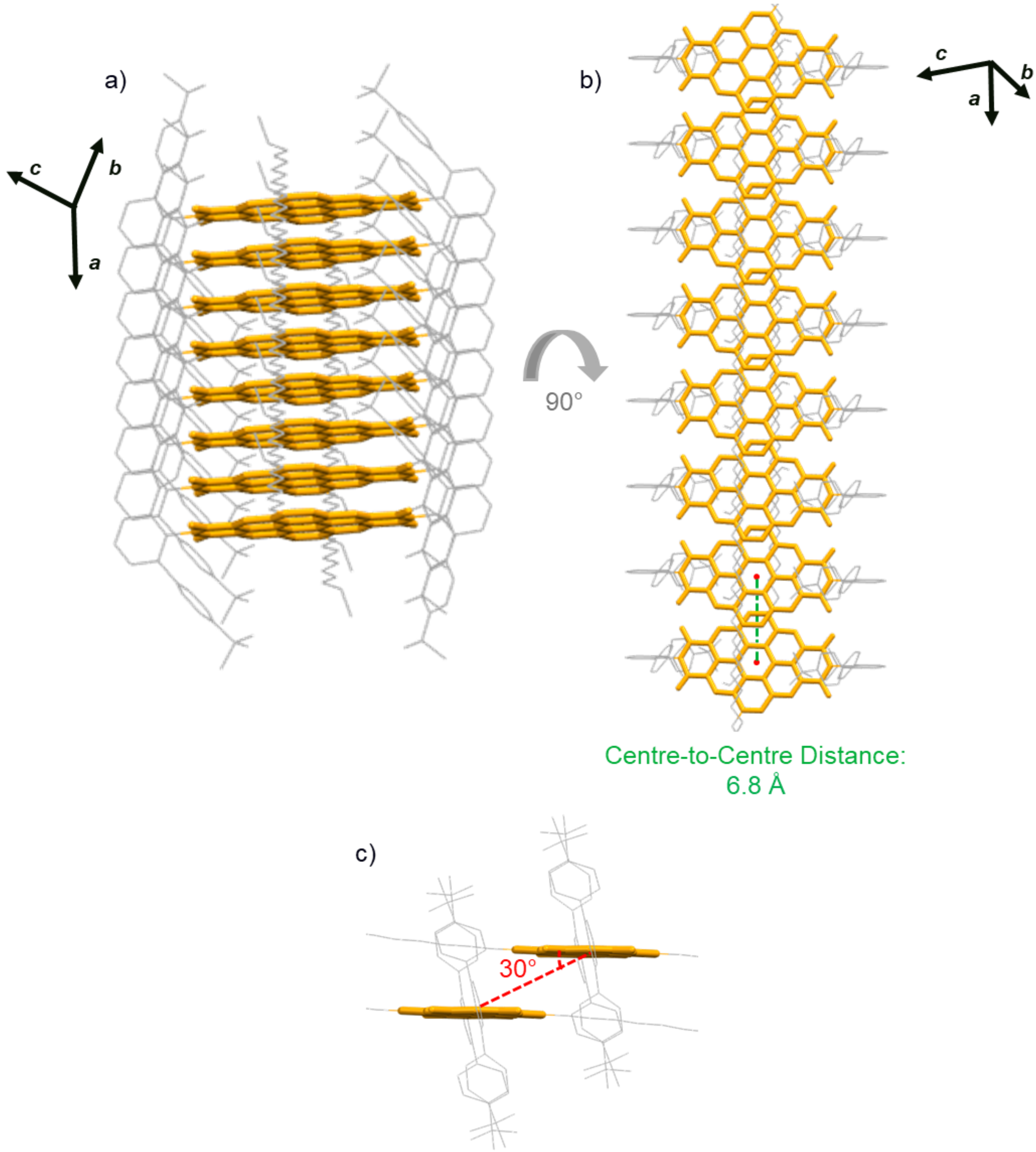

Figure S3. Packing of CBI **1** as extended π-stacks **[1]$_n$** in the single crystal grown from iodobenzene solution (c_0 = 10^{-3} M) by slow diffusion of methanol according to X-ray analysis shown in a) side- and b) top-view. The CBI chromophore (orange) is displayed in capped sticks, whereas the imide substituents and the substituents at the CBI core are displayed in wireframe (grey). The centre-to-centre distance is given in green. c) Side-view onto two π-stacked CBIs showing the angle between the two centres in red. Molecular disorder and solvent molecules (methanol) are omitted for clarity.

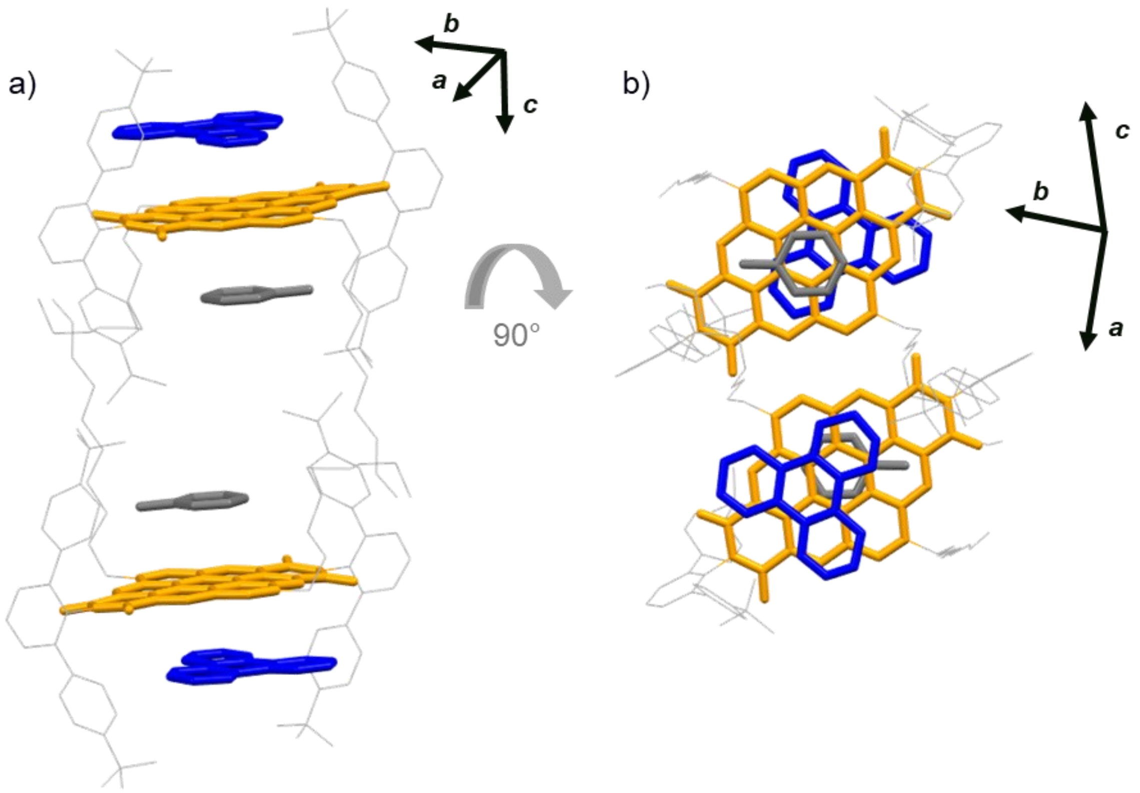

Figure S4. a) Side- and b) top-view of the cocrystal **[1·T]** showing that isolated complexes with **T** (blue) and toluene (grey) on each side of CBI **1** are formed. Single crystals were obtained from a mixture of CBI **1** ($c_0 = 10^{-3}$ M) with **T** (1:2) in toluene by slow diffusion of methanol. The CBI chromophore (orange) and guest molecule **T** are displayed in capped sticks, whereas the imide substituents and the substituents at the CBI core are displayed in wireframe (grey). Molecular disorder is omitted for clarity.

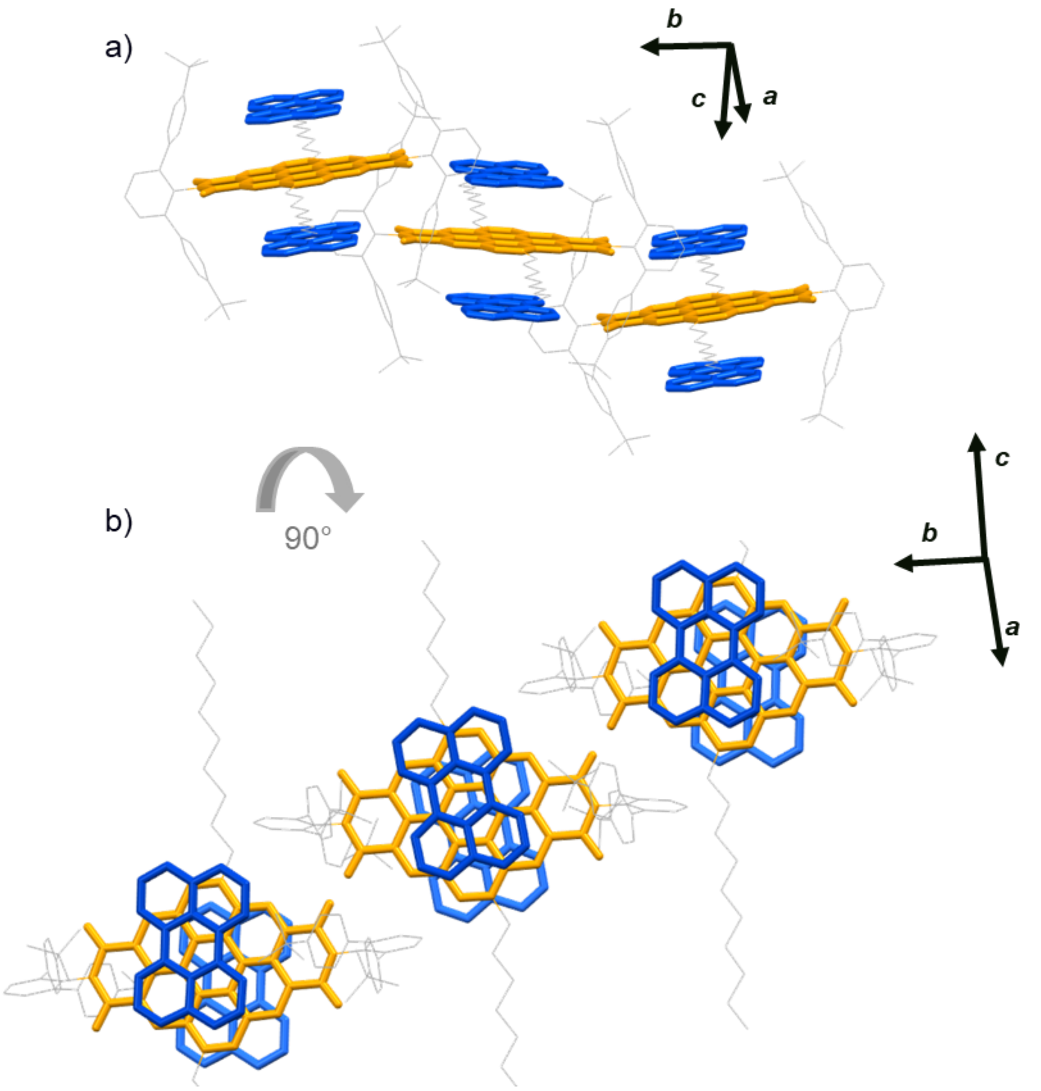

Figure S5. a) Side- and b) top-view of the cocrystal **[P·1·P]** showing that isolated 1:2 complexes are formed. Single crystals were obtained from a mixture of CBI **1** (c_0 = 10^{-3} M) with **P** (1:2) in toluene by slow diffusion of methanol. The CBI chromophore (orange) and guest molecule **P** (blue) are displayed in capped sticks, whereas the imide substituents and the substituents at the CBI core are displayed in wireframe (grey). Molecular disorder is omitted for clarity.

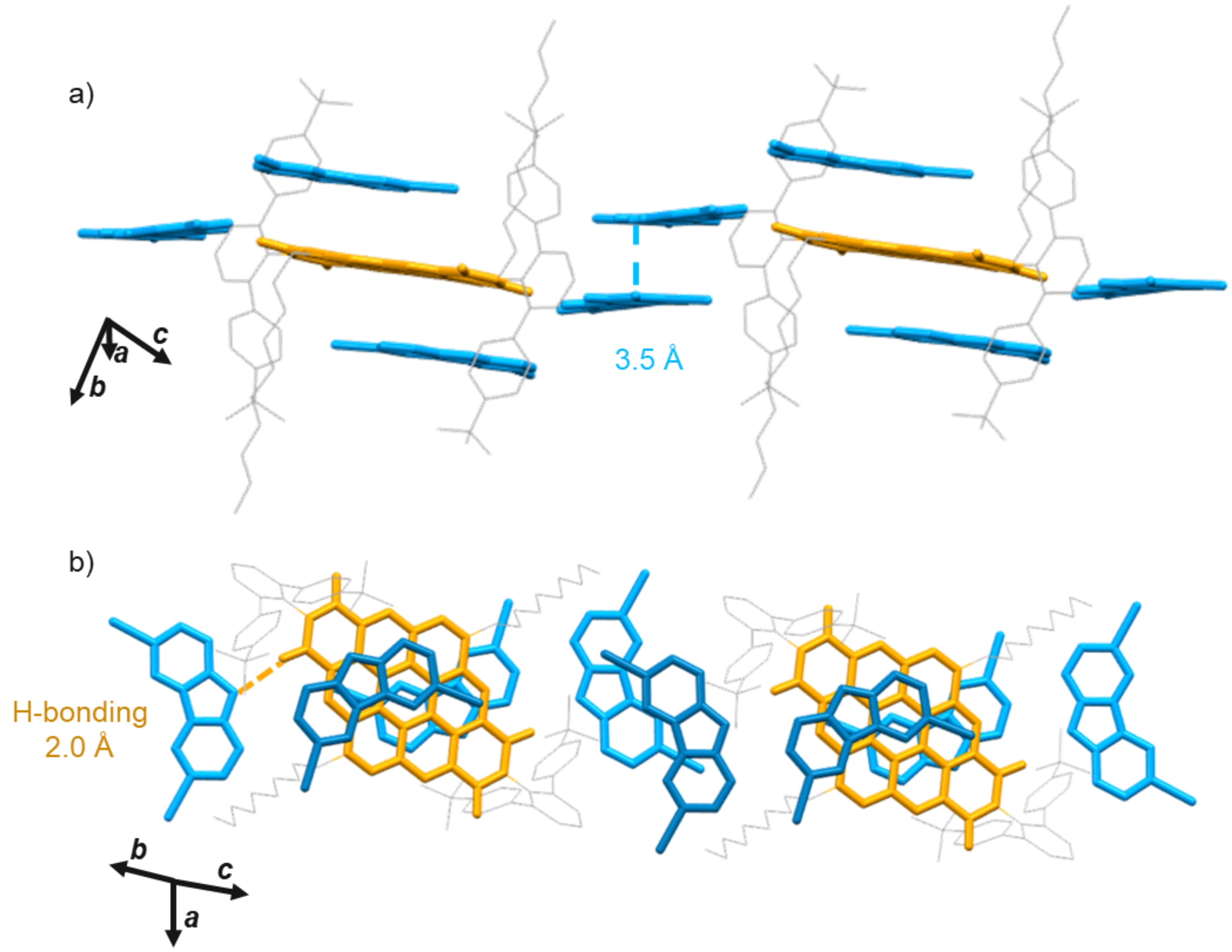

Figure S6. a) Side- and b) top-view of the cocrystal **[I₂Cz·1·I₂Cz]** showing that CBI is surrounded by four **I₂Cz** molecules. The distance of the stacked **I₂Cz** molecules is also shown. Single crystals were obtained from a mixture of CBI **1** ($c_0 = 10^{-3}$ M) with **I₂Cz** (1:4) in $CHCl_3$ by slow diffusion of *n*-hexane. The CBI chromophore (orange) and guest molecule **I₂Cz** (blue) are displayed in capped sticks, whereas the imide substituents and the substituents at the CBI core are displayed in wireframe (grey). Molecular disorder and solvent molecules are omitted for clarity.

6. Aggregation Study

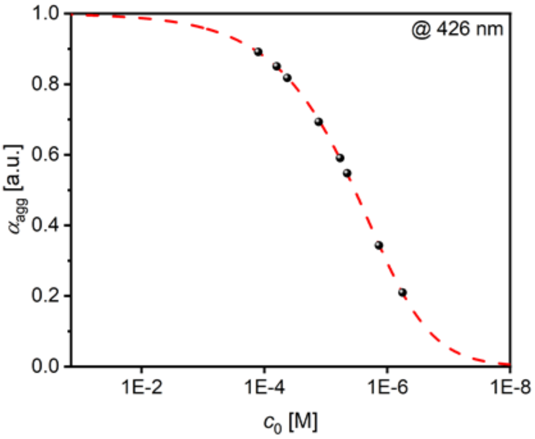

Figure S7. Degree of aggregation α_{Agg} of CBI **1** in MCH/TCE (85/15) at λ_{max} = 426 nm (black symbols) as estimated by the dimer model from the values ε_M and ε_D determined from the global fit analysis.[S19]

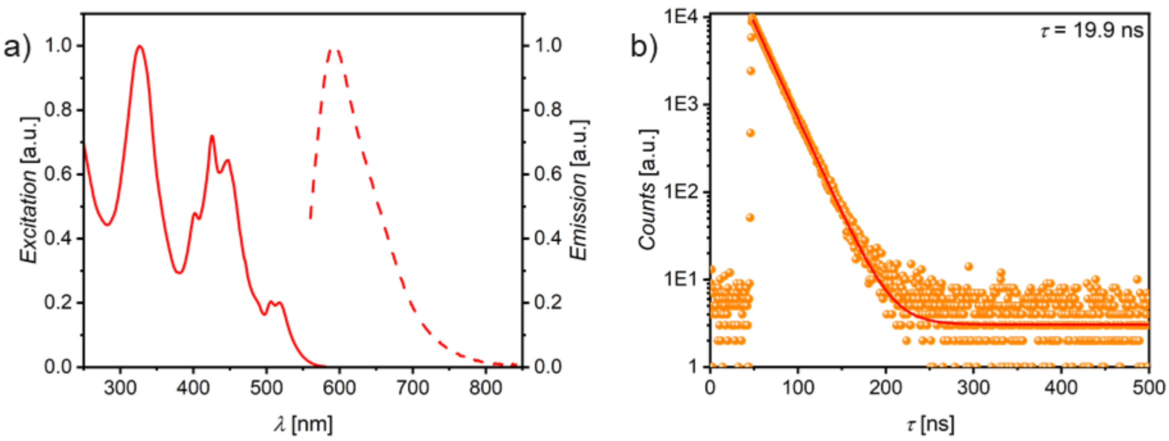

Figure S8. a) Excitation (solid) and emission (dashed) spectra of CBI **1** in MCH/TCE (85/15; $c_0 = 1.26 \times 10^{-4}$ M, α_{Agg} = 89%). The emission spectrum was recorded with an excitation wavelength of 540 nm. b) Lifetime measurement (orange circles) with excitation at 540 nm while detecting at 594 nm and the respective mono-exponential fit (red line).

7. Theoretical Calculation

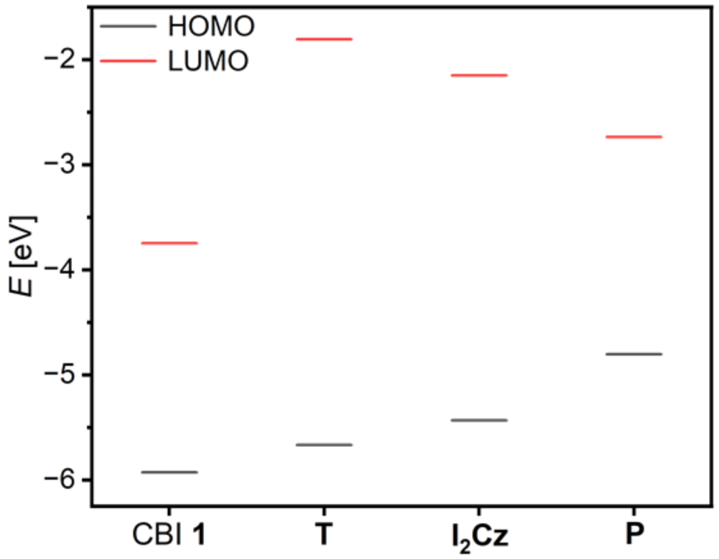

Figure S9. Comparison of the calculated HOMO (black lines) and LUMO (red lines) levels of the CBI **1** and the three guest molecules **T**, **I$_2$Cz** and **P**.

8. Complexation Studies

All UV/Vis absorption spectra of the CBI **1** titration studies with perylene (**P**), triphenylene (**T**) and 3,6-diiodocarbazole (**I_2Cz**) are available in the Zenodo repository DOI: 10.5281/zenodo.19203936.

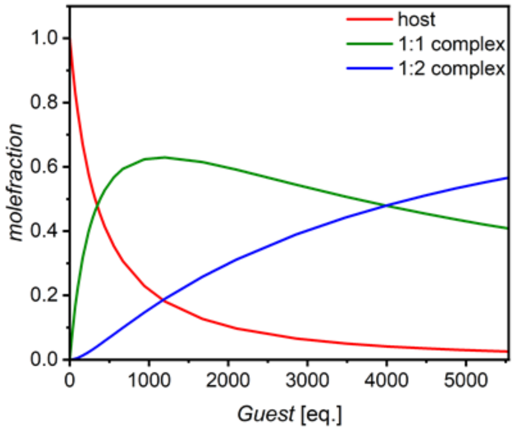

Figure S10. Molar fractions of free host CBI **1** (red), 1:1 complex (green) and 1:2 complex (blue) according to global fit (490−550 nm) analysis in 1:2 model with K_1 = 199 and K_2 = 17 M^{-1} for the UV/Vis complexation study of CBI **1** (c_0(**1**) = 1.45 × 10^{-5} M) with **T** as guest in TCE at 298 K shown in Figure 3b.

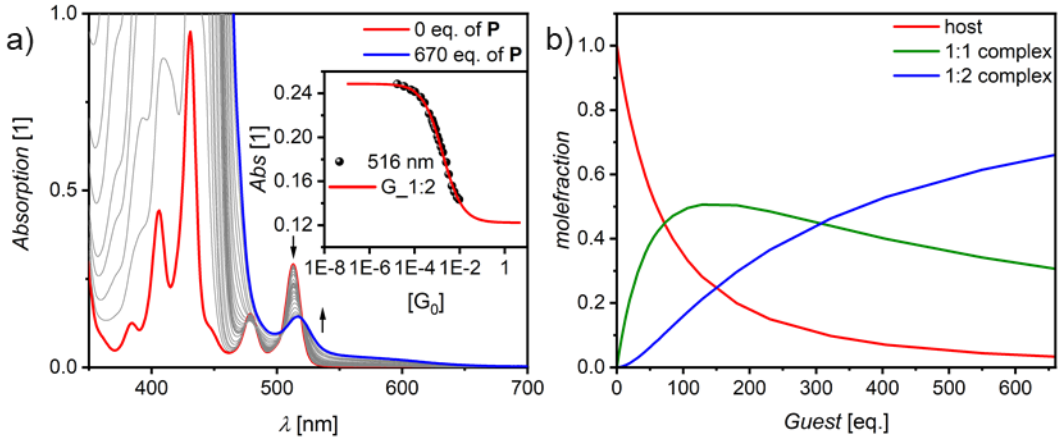

Figure S11. a) UV/Vis absorption spectra (solid lines) for a solution of CBI **1** as host (c_0 = 1.40 × 10^{-5} M, red line) and changes upon addition of **P** as guest (grey to blue lines, 670 eq.) in TCE at 298 K. Arrows depict spectral changes with increasing eq. of the guest **P**. Inset shows the absorption at λ = 516 nm (black symbol) with nonlinear curve to the 1:2 (red line) global (513−600 nm) model. b) Molar fractions of free host (red), 1:1 complex (green) and 1:2 complex (blue) according to global fit (513−600 nm) in 1:2 model with K_1 = 1008 and K_2 = 235 M^{-1}.

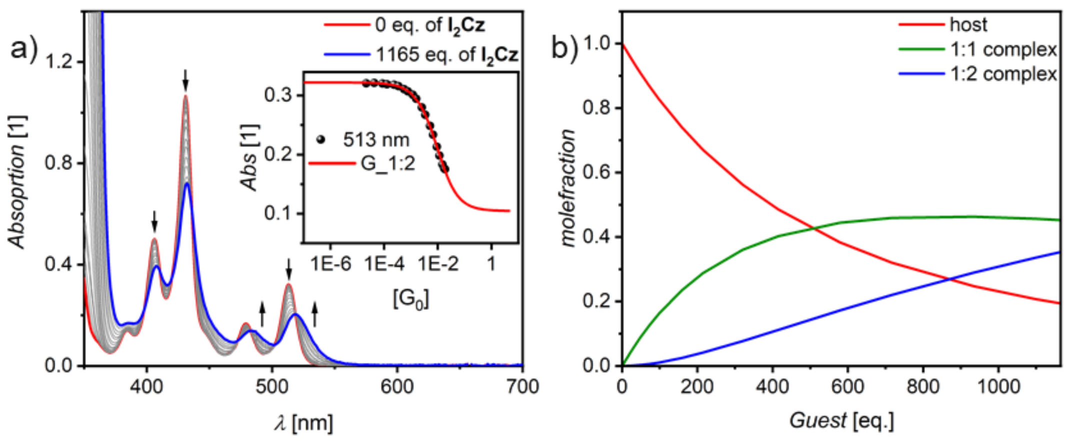

Figure S12. a) UV/Vis absorption spectra (solid lines) for a solution of CBI **1** as host ($c_0 = 1.59 \times 10^{-5}$ M, red line) and changes upon addition of **I₂Cz** as guest (grey to blue lines, 1165 eq.) in TCE at 298 K. Arrows depict spectral changes with increasing eq. of the guest **I₂Cz**. Inset shows the absorption at $\lambda = 513$ nm (black symbol) with nonlinear curve to the 1:2 (red line) global (490−550 nm) model. b) Molar fractions of free host (red), 1:1 complex (green) and 1:2 complex (blue) according to global fit (490−550 nm) in 1:2 model with $K_1 = 126$ and $K_2 = 42$ M^{-1}.

Table S7. Binding constants and Gibbs free binding energies of host CBI **1** and guest molecules **P**, **T** and **I₂Cz** in TCE at 298 K.

**Guest** (Fit)	K_1 [M^{-1}]	K_2 [M^{-1}]	ΔG_1[c] [kJ mol^{-1}]	ΔG_2[c] [kJ mol^{-1}]
**P** (1:2)[a]	1008	235	−17.1	−13.5
**T** (1:2)[b]	199	17	−13.1	−7.05
**I₂Cz** (1:2)[b]	126	42	−12.0	−9.27

Binding constants $K_{1/2}$ determined with the program bindfit using global fit analysis for the 1:2 model; a) range: λ = 513−600 nm; b) range: λ = 490−550 nm; c) Gibbs free energies $\Delta G_{1/2}$(298 K) calculated from $K_{1/2}$ according to $\Delta G_{1/2}(298\text{ K}) = -RT \ln(K_{1/2})$.

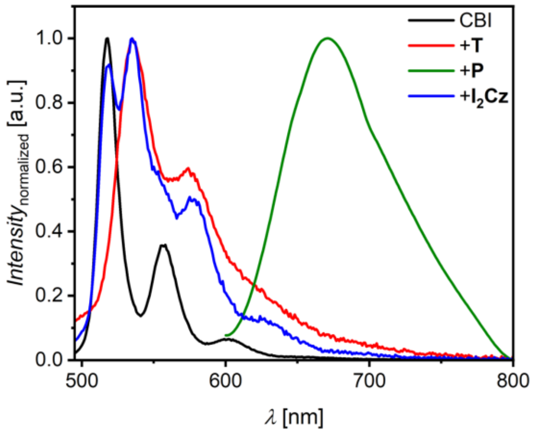

Figure S13. Photoluminescence spectra (solid lines) of a solution of CBI **1** before ($c_0 \sim 5 \times 10^{-7}$ M, black line) and after addition of **T** (red line; 141636 eq.), **P** (green line; 20802 eq.) and **I_2Cz** (blue line; 62978 eq.) in TCE under ambient conditions at 298 K. Emission spectra were recorded with excitation at $\lambda_{ex} = 580$ (**P**) and 482.5 nm (**1**, **T** and **I_2Cz**), respectively.

Table S8. Summary of the optical properties of **1** as a monomer (c_0(**1**) = 2.7×10^{-7} M), dimer (**1_2**; c_0(**1**) = 1.26×10^{-4} M) and upon complexation (c_0(**1**) ~ 5×10^{-7} M) with guest molecules **T** (141636 eq.), **I_2Cz** (62978 eq.) and **P** (20802 eq.) in solution at 298 K under ambient conditions.

**Material**	λ_{abs} [nm]	λ_{em} [nm]	**PL**
**1**[a]	430	516	FL
**1_2**[b]	448	594	EX
**1+T**[c]	440	535	CT
**1+I_2Cz**[c]	431	535	CT
**1+P**[c]	517	676	CT

[a] Measured in $CHCl_3$; [b] Measured in MCH/TCE (85/15); [c] Measured in TCE.

9. X-Ray Diffraction Measurements

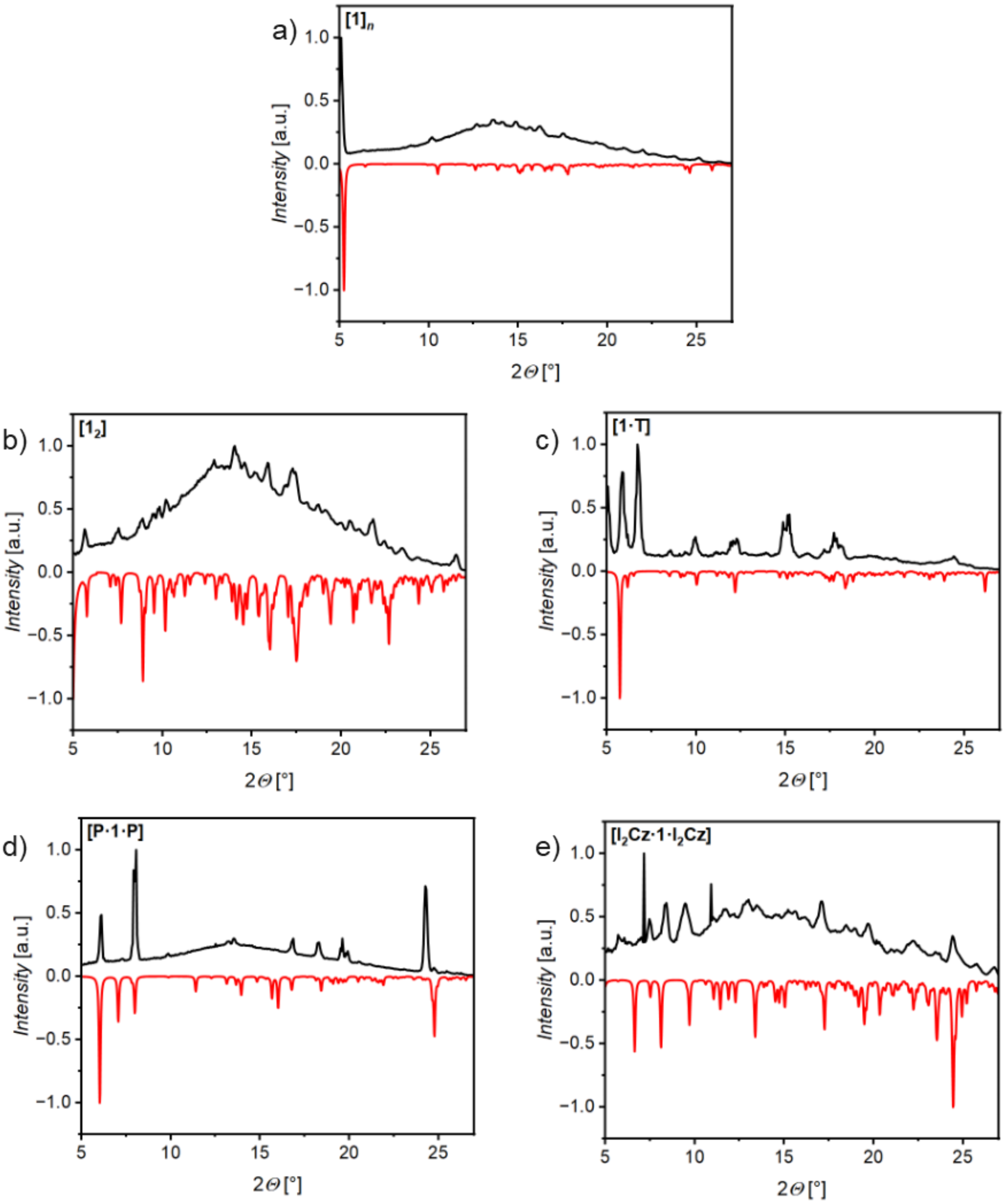

Figure S14. Comparison of the experimental (black) and simulated (red) X-ray diffraction pattern of the a) **[1]**$_n$, b) **[1$_2$]**, c) **[1·T]**, d) **[P·1·P]** and e) **[I$_2$Cz·1·I$_2$Cz]** crystals.

10. PL Measurement of Crystals

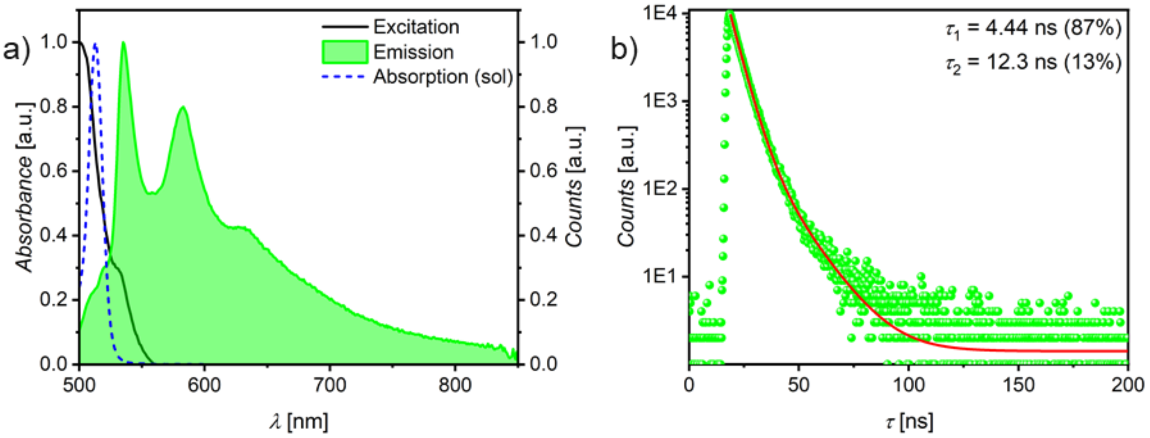

Figure S15. a) PL measurement of an ensemble of crystals of **[1]**$_n$ on Si/SiO_2 substrate at room temperature under ambient conditions. Normalized excitation (black line, λ_{em} = 580 nm) and emission (green, λ_{ex} = 440 nm) spectra of the crystals as well as the absorption of **1** (dashed blue line) in $CHCl_3$ solution are shown. b) PL decay curves (green circles) at room temperature while detecting at their respective emission maxima at 537 nm during excitation at λ_{ex} = 440 nm as well as the best fit (red line). The lifetime components of the PL decay are given as well.

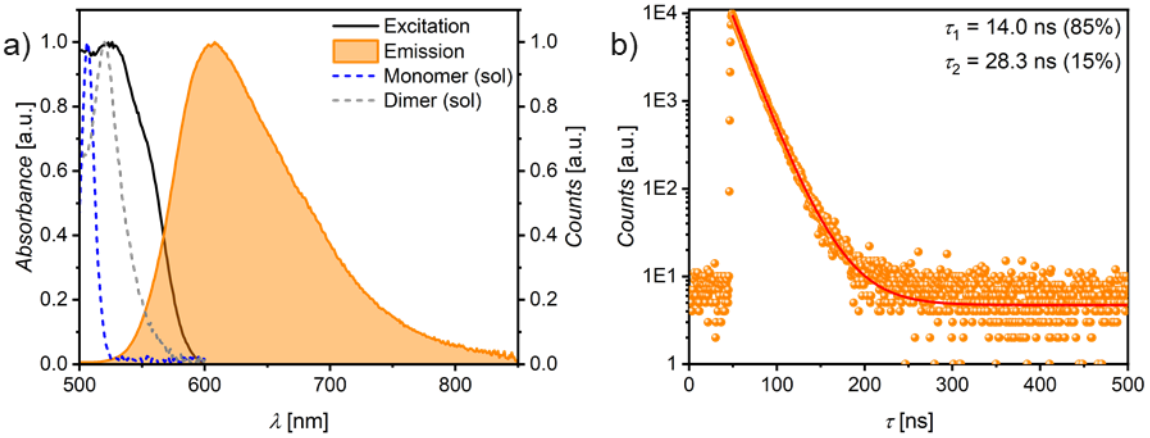

Figure S16. a) PL measurement of an ensemble of crystals of **[1$_2$]** on Si/SiO_2 substrate at room temperature under ambient conditions. Normalized excitation (black line, λ_{em} = 620 nm) and emission (orange, λ_{ex} = 440 nm) spectra of the crystals as well as the calculated monomer (dashed blue line) and dimer (dashed grey line) absorption of **1** in MCH/TCE (85/15) solution are shown. b) PL decay curves (orange circles) at room temperature while detecting at their respective emission maxima at 608 nm during excitation at λ_{ex} = 440 nm as well as the best fit (red line). The lifetime components of the PL decay are given as well.

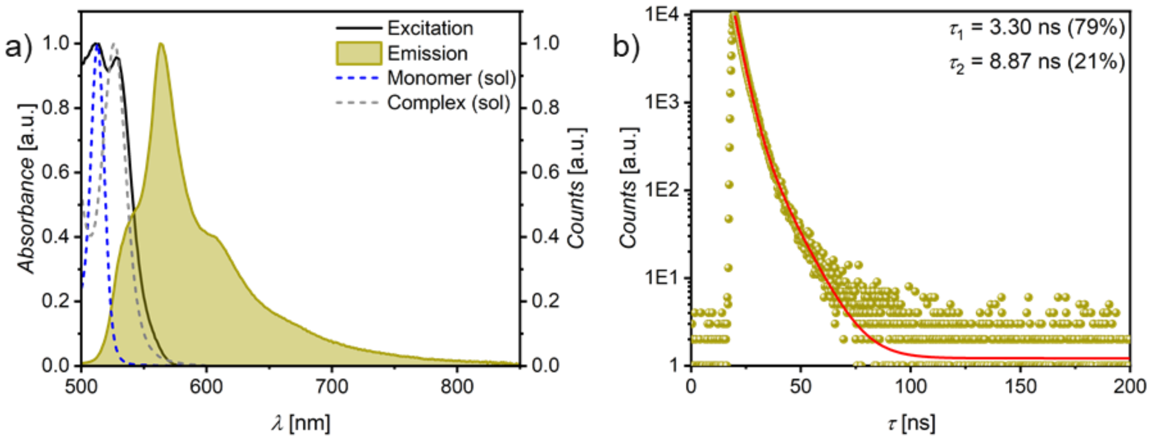

Figure S17. a) PL measurement of an ensemble of cocrystals of **[1·T]** on Si/SiO_2 substrate at room temperature under ambient conditions. Normalized excitation (black line, λ_{em} = 600 nm) and emission (yellow, λ_{ex} = 476 nm) spectra of the cocrystals as well as the absorption of **1** before (dashed blue line) and after the addition of **T** (dashed grey line; 5538 eq.) in TCE solution are shown. b) PL decay curves (yellow circles) at room temperature while detecting at their respective emission maxima at 565 nm during excitation at λ_{ex} = 440 nm as well as the best fit (red line). The lifetime components of the PL decay are given as well.

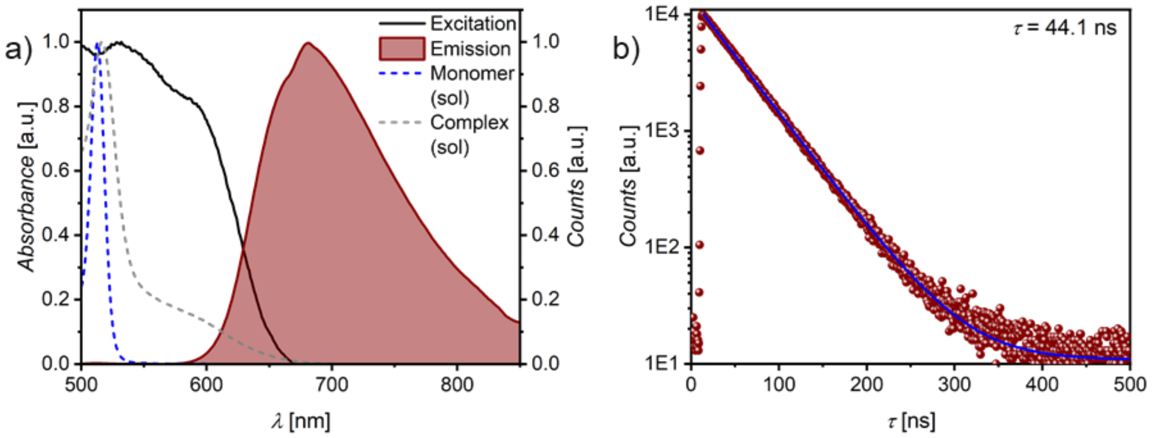

Figure S18. a) PL measurement of an ensemble of cocrystals of **[P·1·P]** on Si/SiO_2 substrate at room temperature under ambient conditions. Normalized excitation (black line, λ_{em} = 690 nm) and emission (wine red, λ_{ex} = 476 nm) spectra of the cocrystals as well as the absorption of **1** before (dashed blue line) and after the addition of **P** (dashed grey line; 670 eq.) in TCE solution are shown. b) PL decay curves (wine red circles) at room temperature while detecting at their respective emission maxima at 681 nm during excitation at λ_{ex} = 476 nm as well as the best fit (blue line). The lifetime components of the PL decay are given as well.

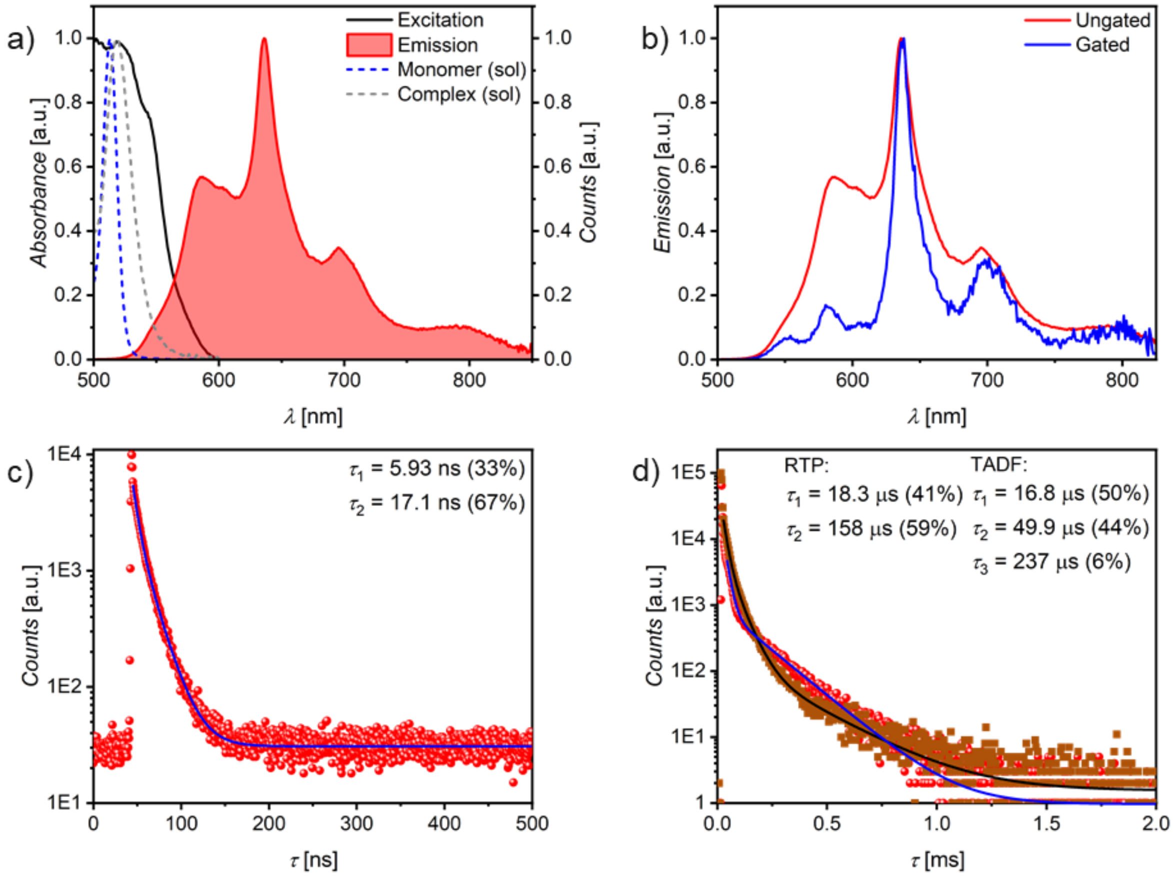

Figure S19. a) PL measurement of an ensemble of cocrystals of **[I_2Cz·1·I_2Cz]** on Si/SiO_2 substrate at room temperature under ambient conditions. Normalized excitation (black line, $\lambda_{em} = 620$ nm) and emission (red, $\lambda_{ex} = 452$ nm) spectra of the cocrystals as well as the absorption of **1** before (dashed blue line) and after the addition of **I_2Cz** (dashed grey line; 1165 eq.) in TCE solution are shown. b) Comparison of the ungated (red line) and gated (blue line) emission spectra of **[I_2Cz·1·I_2Cz]**. c) PL decay curves (red circles) at room temperature while detecting at 587 nm during excitation at $\lambda_{ex} = 479.7$ nm as well as the best fit (blue line). d) PL decay curves at room temperature while detecting either the respective emission maximum at 635 nm (red circles) or at 587 nm (brown squares) during excitation at $\lambda_{ex} = 452$ nm as well as their best fits (blue and black lines). The lifetime components of the PL decays are given as well.

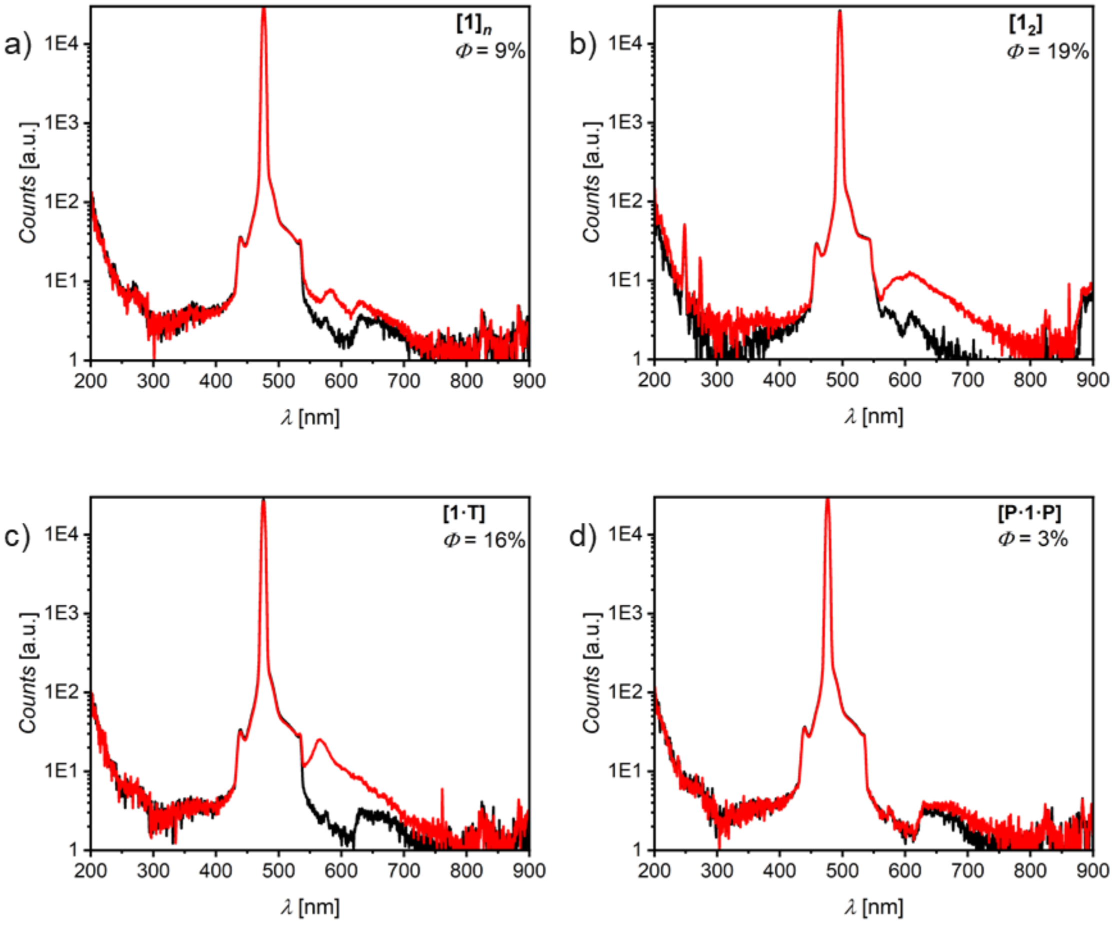

Figure S20. Representative optical profiles of an integrating sphere measurements for the determination of Φ_{PL} of reference (black line) and an ensemble of single crystals (red line) of a) **[1]** $_n$, b) **[1₂]**, c) **[1·T]** and d) **[P·1·P]** on quartz substrate at 298 K upon excitation at λ_{ex} = 476 nm.

11. Calculations for Exciton Coupling in [1]$_n$ and [1$_2$] Crystals

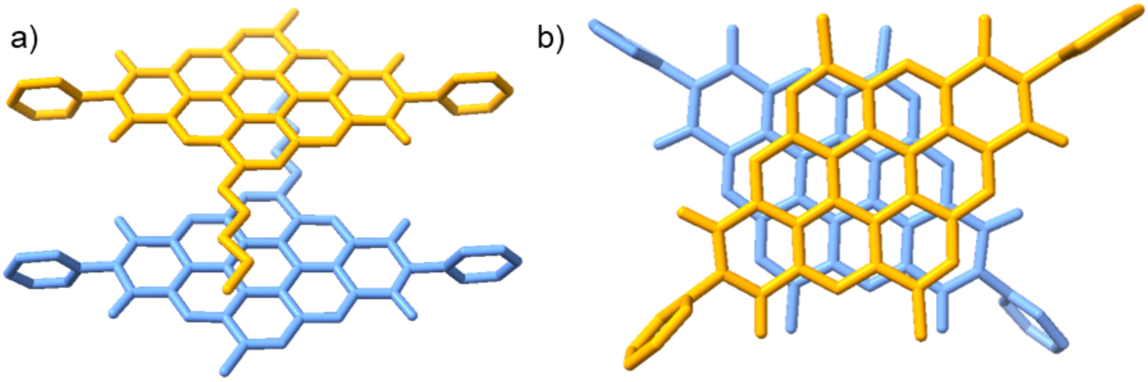

Figure S21. Dimer structures used for theoretical calculations of a) **[1]$_n$** and b) **[1$_2$]**.

Table S9. Calculated exciton coupling energies for dimer pairs of **[1]$_n$** and **[1$_2$]**.

		[1]$_n$	[1$_2$]
J_{total}	cm^{-1}	−94	471
J_{Coul}	cm^{-1}	−18	−101
J_{CT}	cm^{-1}	−75	573

12. Polarization-dependence of Single- and Cocrystals

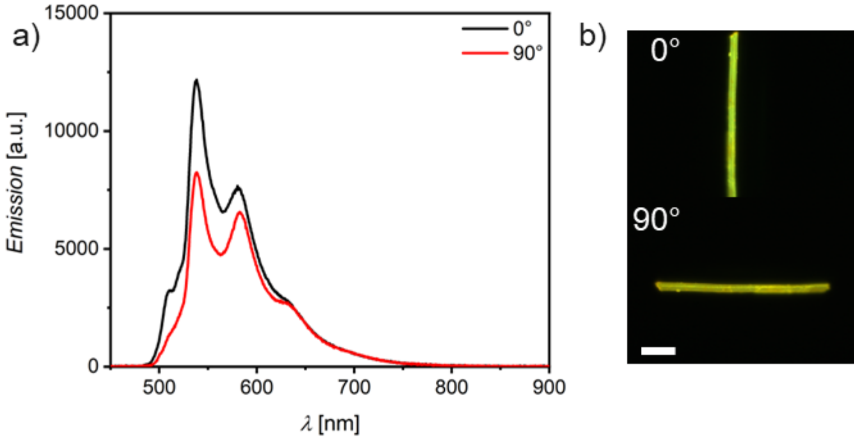

Figure S22. a) Polarization-dependent emission spectra of **[1]*n*** at 0° (black) and 90° (red) polarization with respect to the long crystal axis. b) Microscopic pictures of the cocrystal with analyzer at 0° and 90°. The scale bars equal each 100 µm.

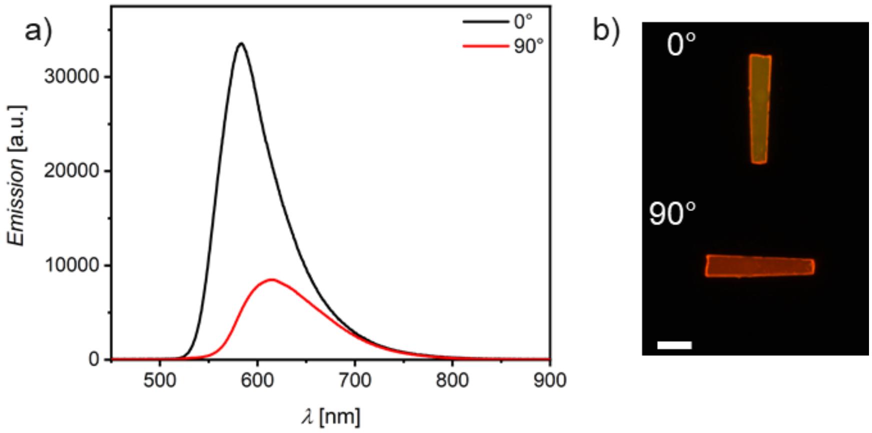

Figure S23. a) Polarization-dependent emission spectra of **[1₂]** at 0° (black) and 90° (red) polarization with respect to the long crystal axis. b) Microscopic pictures of the cocrystal with analyzer at 0° and 90°. The scale bars equal each 100 µm.

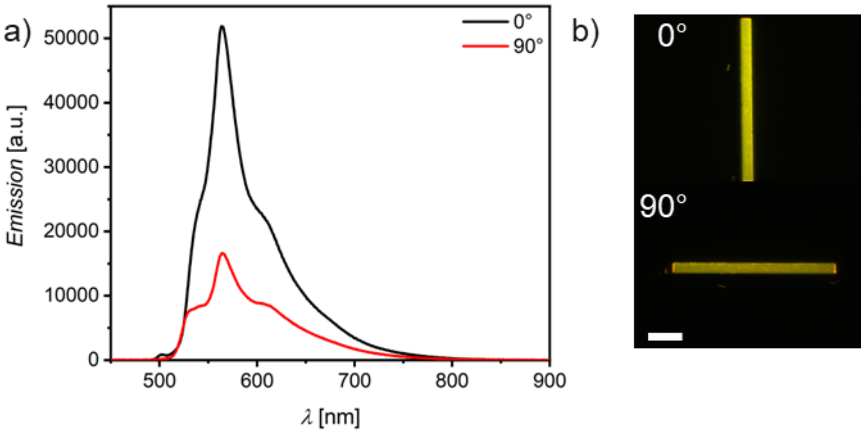

Figure S24. a) Polarization-dependent emission spectra of **[1·T]** at 0° (black) and 90° (red) polarization with respect to the long crystal axis. b) Microscopic pictures of the cocrystal with analyzer at 0° and 90°. The scale bars equal each 100 µm.

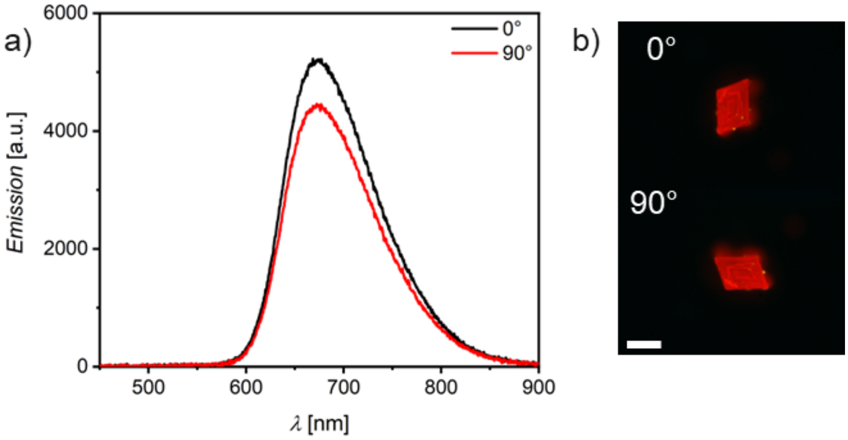

Figure S25. a) Polarization-dependent emission spectra of **[P·1·P]** at 0° (black) and 90° (red) polarization with respect to the long crystal axis. b) Microscopic pictures of the cocrystal with analyzer at 0° and 90°. The scale bars equal each 100 µm.

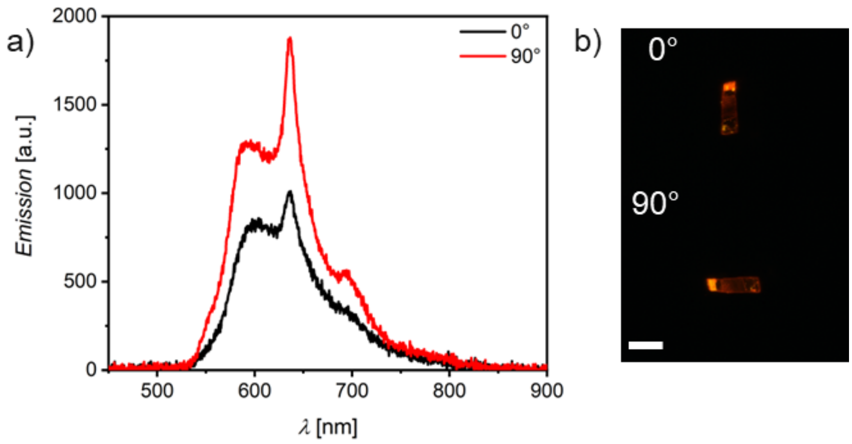

Figure S26. a) Polarization-dependent emission spectra of **[I_2Cz·1·I_2Cz]** at 0° (black) and 90° (red) polarization with respect to the long crystal axis. b) Microscopic pictures of the cocrystal with analyzer at 0° and 90°. The scale bars equal each 100 µm.

13. Temperature-dependent PL Measurement

Table S10. Temperature-dependent photoluminescent parameters of **[I_2Cz·1·I_2Cz]** cocrystals on a Si/SiO_2 substrate.

T [K]	*τ* [μs]	*Rel.* [%]	χ^2 [1]
298	18.3<br>158.1	41<br>59	1.475
280	16.9<br>165.4<br>453.0	26<br>35<br>39	1.387
260	16.5<br>262.7<br>991.9	17<br>31<br>51	1.444
240	86.9<br>851.3<br>2385.9	12<br>46<br>43	1.026
220	239.4<br>1429.0<br>3906.3	9<br>43<br>48	0.810
200	233.2<br>1559.8<br>4802.4	8<br>40<br>52	0.879
180	292.0<br>1745.0<br>5265.4	10<br>42<br>48	1.000
160	324.7<br>1805.7<br>5437.6	11<br>43<br>47	1.059
140	420.5<br>1925.5<br>5495.2	11<br>43<br>47	1.160
120	371.0<br>1917.3<br>5411.9	11<br>44<br>44	1.076
100	317.9<br>1729.5<br>5090.3	8<br>42<br>50	1.044
80	353.5<br>1800.8<br>5054.9	7<br>40<br>53	0.975

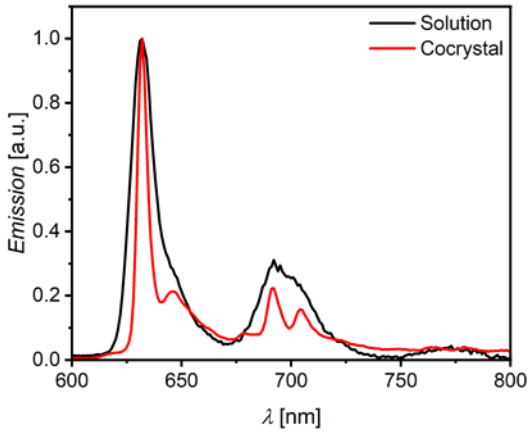

Figure S27. Low-temperature PL measurements for CBI **1** with ethyl iodide (black, 2 mL) in $CHCl_3$ ($c_0 = 1 \times 10^{-5}$ M) at 80 K as well as the respective PL spectra of cocrystals with **I₂Cz** on a Si/SiO_2 substrate at 80 K (red).

14. NMR Spectroscopy

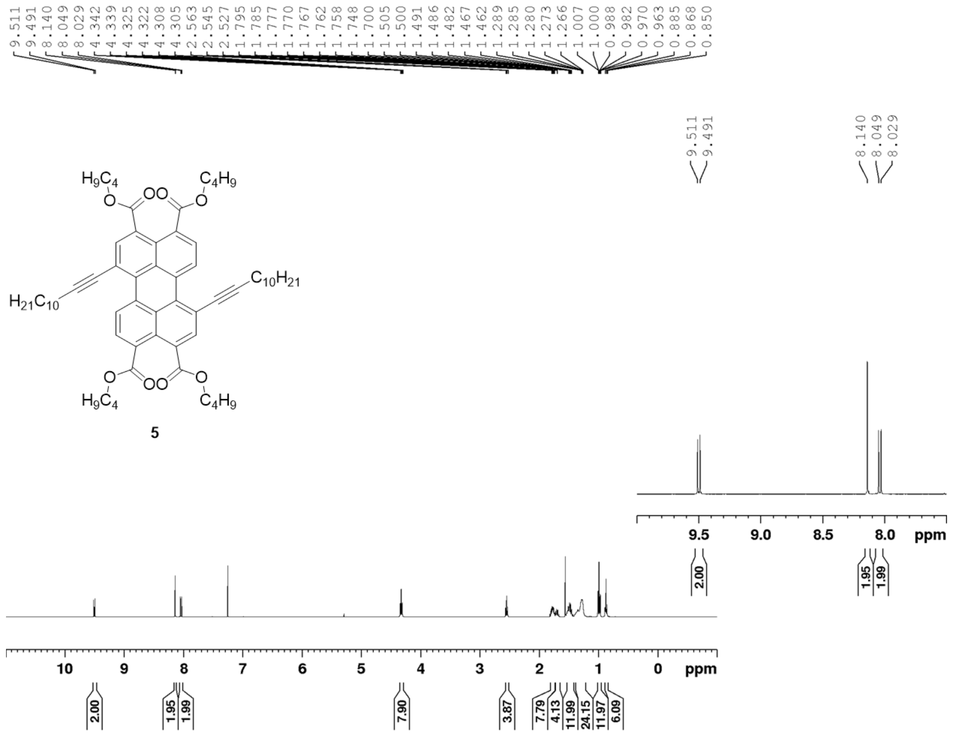

Figure S28. ^{1}H-NMR spectrum (400 MHz) of **5** in $CDCl_3$ at 295 K.

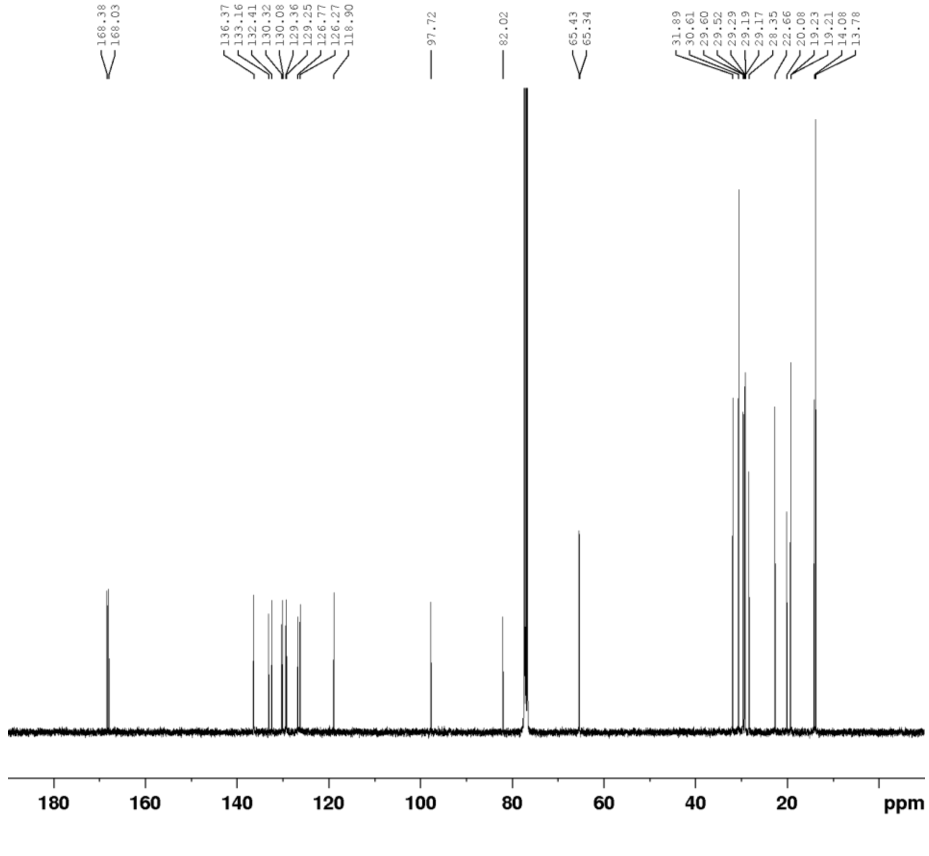

Figure S29. ^{13}C-NMR spectrum (101 MHz) of **5** in $CDCl_3$ at 295 K.

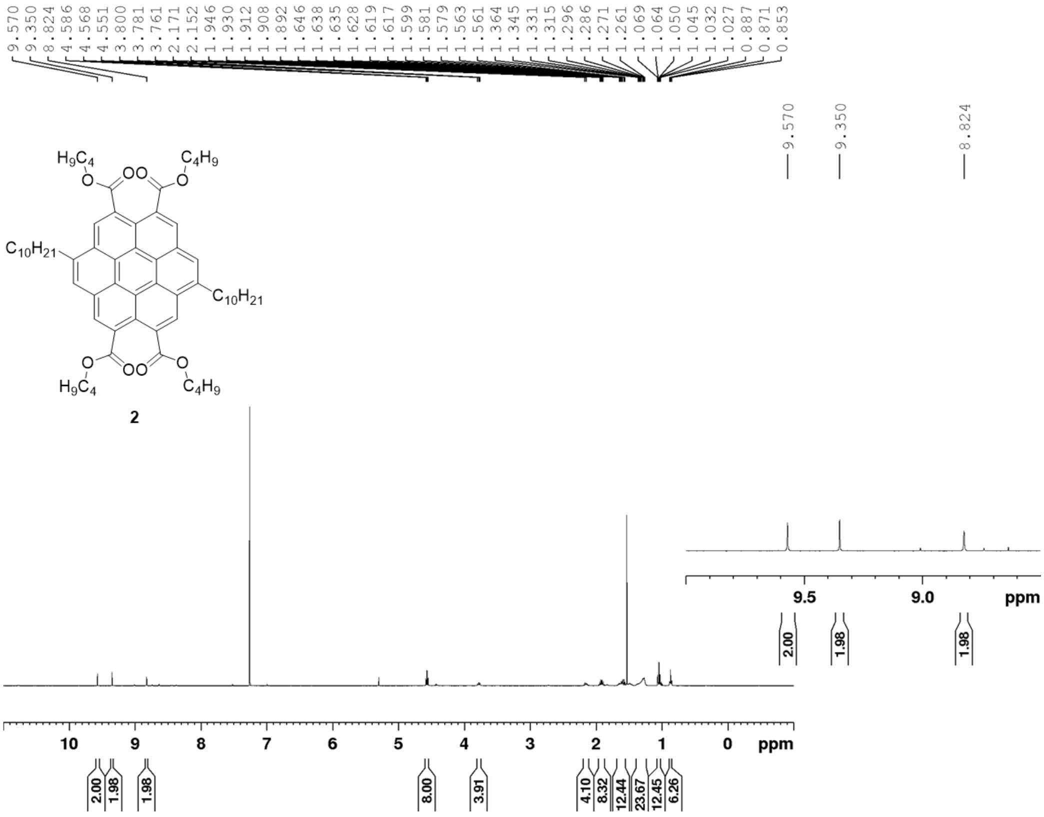

Figure S30. ^{1}H-NMR spectrum (400 MHz) of **2** in $CDCl_3$ at 295 K.

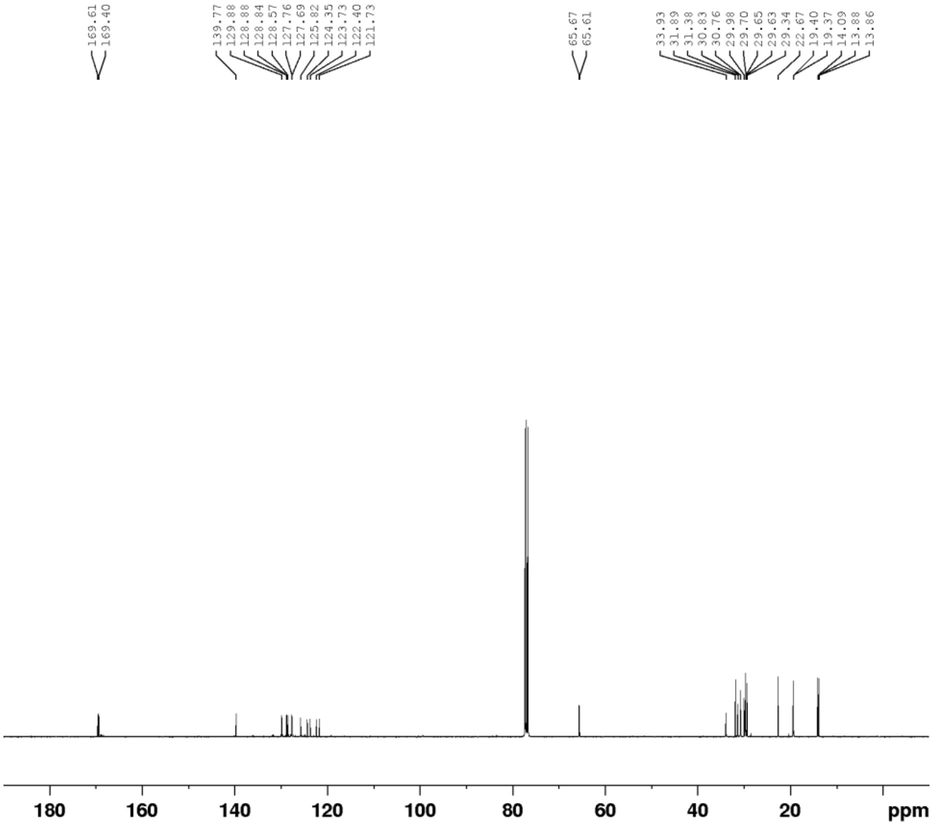

Figure S31. ^{13}C-NMR spectrum (101 MHz) of **2** in $CDCl_3$ at 295 K.

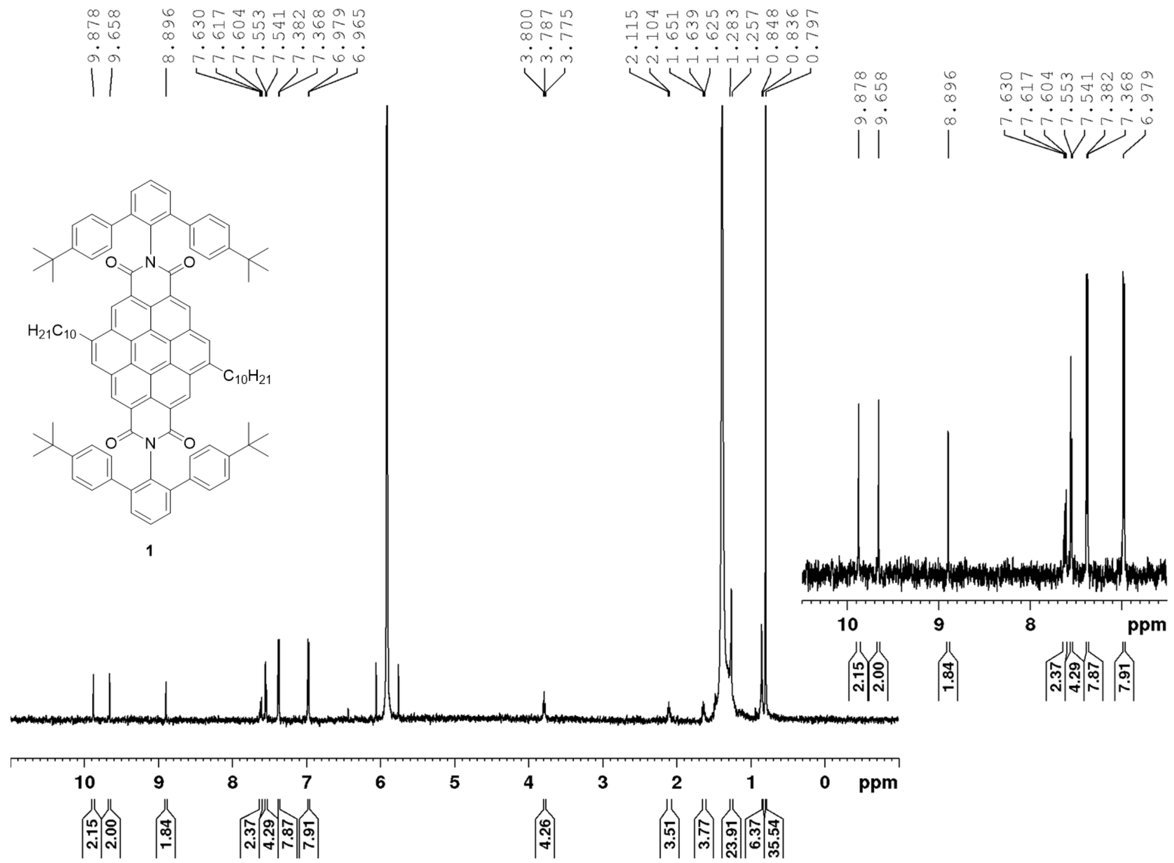

Figure S32. ^{1}H-NMR spectrum (600 MHz) of CBI **1** in $C_2D_2Cl_4$ at 374 K.

15. Mass Spectrometry

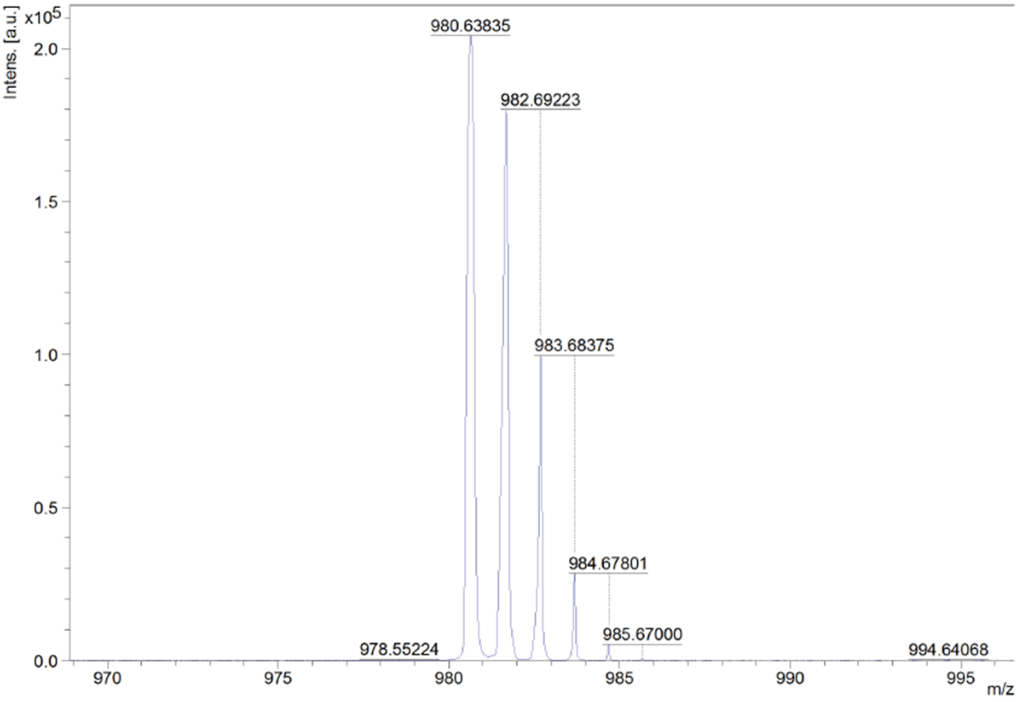

Figure S33. High-resolution mass spectrum (MALDI-TOF, neg. mode, matrix: DCTB 1:3 in DCM) of **5**.

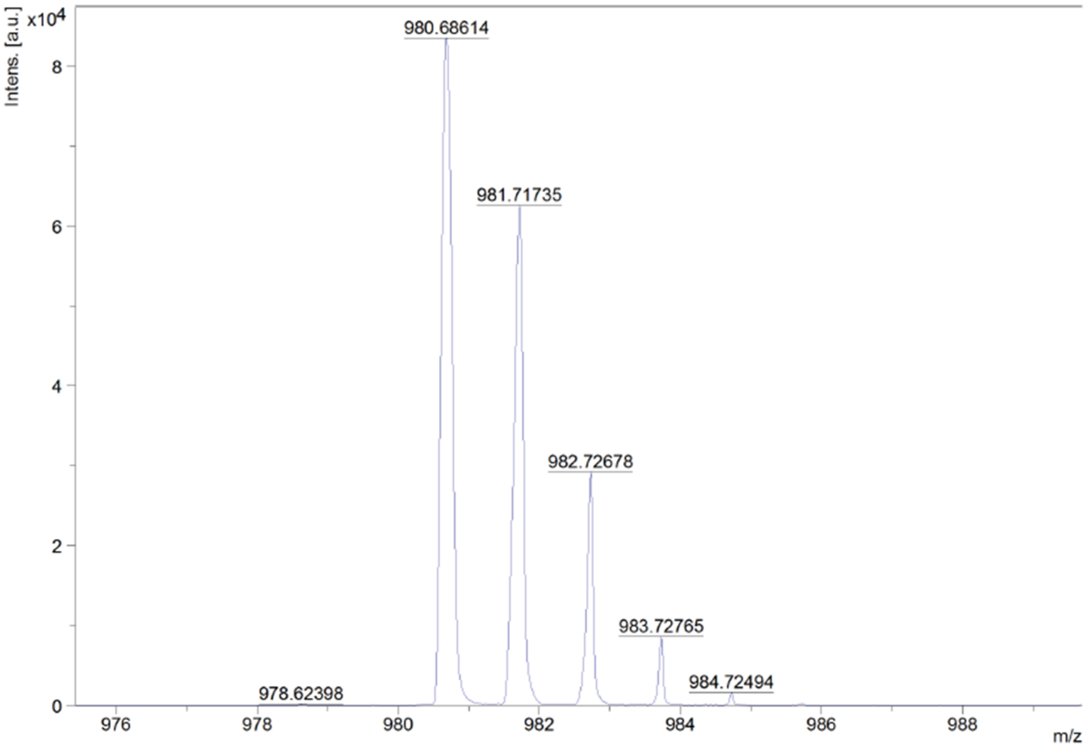

Figure S34. High-resolution mass spectrum (MALDI-TOF, pos. mode, matrix: DCTB 1:3 in DCM) of **2**.

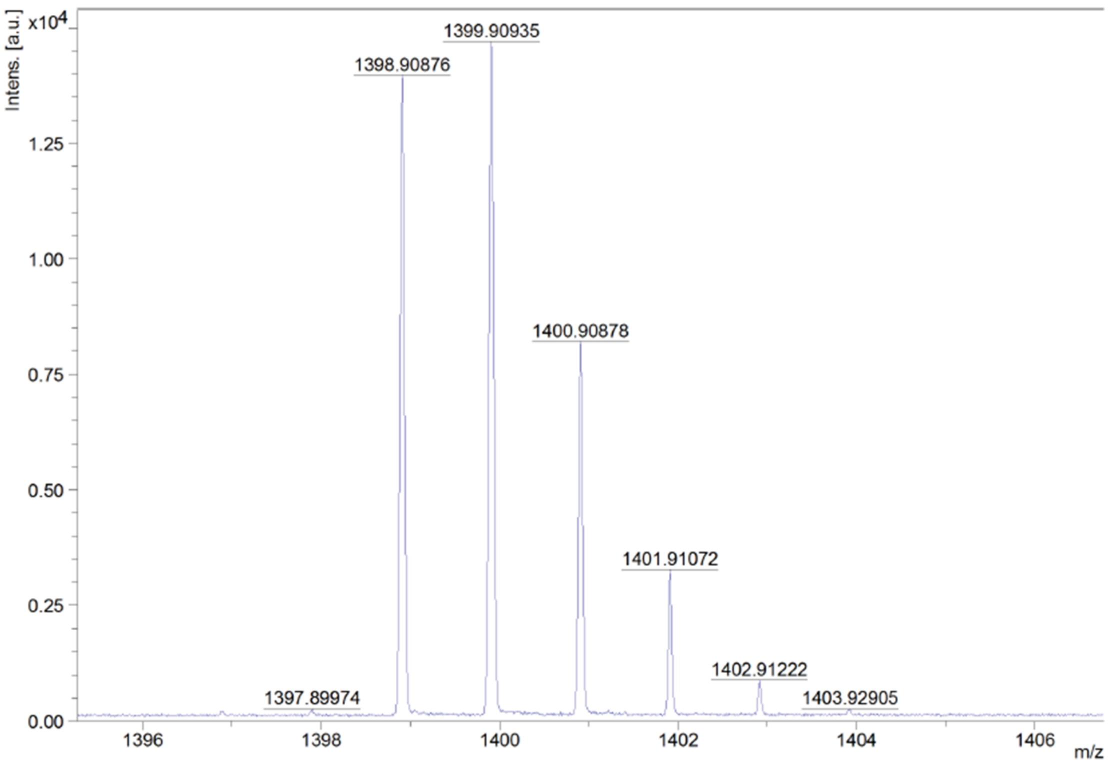

Figure S35. High-resolution mass spectrum (MALDI-TOF, pos. mode, matrix: DCTB 1:3 in DCM) of CBI **1**.

16. References

[S1] M. Mahl, K. Shoyama, A.-M. Krause, D. Schmidt and F. Würthner, *Angew. Chem. Int. Ed.*, 2020, **59**, 13401-13405. DOI: doi.org/10.1002/anie.202004965.

[S2] S. Soldner, O. Anhalt, M. B. Sárosi, M. Stolte and F. Würthner, *Chem. Commun.*, 2023, **59**, 11656-11659. DOI: 10.1039/d3cc03704e.

[S3] S. Brenet, F. Berthiol and J. Einhorn, Eur. *J. Org. Chem.*, 2013, 8094-8096. DOI: 10.1002/ejoc.201301329.

[S4] S. Sengupta, R. K. Dubey, R. W. M. Hoek, S. P. P. van Eeden, D. D. Gunbas, F. C. Grozema, E. J. R. Sudhölter and W. F. Jager, *J. Org. Chem.*, 2014, **79**, 6655-6662. DOI: dx.doi.org/10.1021/jo501180a.

[S5] Z. Zhu, J. Xu, C. Chueh, H. Liu, Z. Li, X. Li, H. Chen and A. Jen, *Adv. Mater.*, 2016, **28**, 10786-10793. DOI: 10.1002/adma.201601745.

[S6] Bindfit (Supramolecular, 2020); supramolecular.org, accessed March 2026.

[S7] P. Thordarson, *Chem. Soc. Rev.*, 2011, **40**, 1305−1323. DOI: 10.1039/C0CS00062K.

[S8] T.-S. Ahn, R. O. Al-Kaysi, A. M. Müller, K. M. Wentz, C. J. Bardeen, *Rev. Sci. Instrum.*, 2007, **78**, 086105. DOI: https://doi.org/10.1063/1.2768926.

[S9] W. Kabsch, *Acta Crystallogr. Sect. D*, 2010, **66**, 125−132. DOI: 10.1107/S0907444909047337.

[S10] G. M. Sheldrick, *Acta Crystallogr. Sect. A*, 2015, **71**, 3−8. DOI: 10.1107/S2053273314026370.

[S11] G. M. Sheldrick, *Acta Crystallogr. Sect. A*, 2008, **64**, 112−122. DOI: 10.1107/S0108767307043930.

[S12] A. L. Spek, *Acta Crystallogr. Sect. C*, 2015, **71**, 9−18. DOI: 10.1107/S2053229614024929.

[S13] A. L. Spek, *Acta Crystallogr. Sect. D*, 2009, **65**, 148−155. DOI: 10.1107/S090744490804362X.

[S14] T. C. Huang , H. Toraya, T. N. Blanton, Y. Wu, *J. Appl. Cryst.*, 1993, **26**, 180–184. DOI: 10.1107/S0021889892009762

[S15] T. N. Blanton, T. C. Huang, H. Toraya, C. R. Hubbard, S. B. Robie, D. Louër, H. E. Göbel, G. Will, R. Gilles, T. Raftery, *Powder Diffr.*, 1995, **10**(2), 91–95. DOI: 10.1017/S0885715600014421.

[S16] P. A. Heiney, Datasqueeze, Version 3.0.23, Pennsylvania, 2023.

[S17] F. Neese, *WIRES Comput. Molec. Sci.*, 2025, **15**, e70019. DOI: 10.1002/wcms.70019.

[S18] M. J. Frisch, G. W. Trucks, H. B. Schlegel, G. E. Scuseria, M. A. Robb, J. R. Cheeseman, G. Scalmani, V. Barone, B. Mennucci, G. A. Petersson, H. Nakatsuji, M. Caricato, X. Li, H. P. Hratchian, A. F. Izmaylov, J. Bloino, G. Zheng, J. L. Sonnenberg, M. Hada, M. Ehara, K. Toyota, R. Fukuda, J. Hasegawa, M. Ishida, T. Nakajima, Y. Honda, O. Kitao, H. Nakai, T. Vreven, J. A. Montgomery, Jr., J. E. Peralta, F. Ogliaro, M. Bearpark, J. J. Heyd, E. Brothers, K. N. Kudin, V. N. Staroverov, T. Keith, R. Kobayashi, J. Normand, K. Raghavachari, A. Rendell, J. C. Burant, S. S. Iyengar, J. Tomasi, M. Cossi, N. Rega, J. M. Millam, M. Klene, J. E. Knox, J. B. Cross, V. Bakken, C. Adamo, J. Jaramillo, R. Gomperts, R. E. Stratmann, O. Yazyev, A. J. Austin, R. Cammi, C. Pomelli, J. W. Ochterski, R. L. Martin, K. Morokuma, V. G. Zakrzewski, G. A. Voth, P. Salvador, J. J. Dannenberg, S. Dapprich, A. D. Daniels, O. Farkas, J. B. Foresman, J. V. Ortiz, J. Cioslowski, D. J. Fox, Gaussian, Inc., Wallingford CT, 2013.

[S19] Y. Vonhausen, F. Würthner, *Chem. Eur. J.*, 2023, **29**, e202300359. DOI: doi.org/10.1002/chem.202300359.
